# Insights on the crosstalk among different cell death mechanisms

**DOI:** 10.1038/s41420-025-02328-9

**Published:** 2025-02-10

**Authors:** Georgette Eskander, Sherihan G. Abdelhamid, Sara A. Wahdan, Sara M. Radwan

**Affiliations:** 1 Postgraduate program, Faculty of Pharmacy, Ain Shams University, Cairo, Egypt; 2 Biochemistry Department, Faculty of Pharmacy, Ain Shams University, Cairo, Egypt; 3 Pharmacology and toxicology Department, Faculty of Pharmacy, Ain Shams University, Cairo, Egypt

**Keywords:** Biochemistry, Cancer

## Abstract

The phenomenon of cell death has garnered significant scientific attention in recent years, emerging as a pivotal area of research. Recently, novel modalities of cellular death and the intricate interplay between them have been unveiled, offering insights into the pathogenesis of various diseases. This comprehensive review delves into the intricate molecular mechanisms, inducers, and inhibitors of the underlying prevalent forms of cell death, including apoptosis, autophagy, ferroptosis, necroptosis, mitophagy, and pyroptosis. Moreover, it elucidates the crosstalk and interconnection among the key pathways or molecular entities associated with these pathways, thereby paving the way for the identification of novel therapeutic targets, disease management strategies, and drug repurposing.

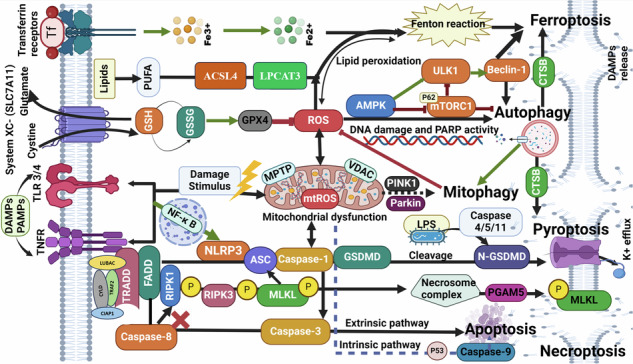

## Facts

- Cell death is crucial for maintaining natural development and growth regulation in organisms.
- The association of many diseases with specific types of programmed cell death (PCD) has facilitated the treatment of certain conditions through the discovery of cell death regulators.
- Recently, crosstalk between different modes of PCD has been elucidated, showing how one type can positively or negatively influence another. This provides new insights into potential treatments for intractable diseases.

## Questions

- How do the signaling pathways of autophagic cell death intersect with those of apoptosis, and what molecular switches determine the cell’s fate towards one pathway over the other?
  - Can modulating the crosstalk between different programmed cell death mechanisms enhance the efficacy of treatments for inflammatory diseases?
  - In what ways does mitophagy contribute to the regulation and execution of different cell death modalities, and how does their interplay affect cellular homeostasis and stress responses?
  - How does oxidative stress influence the crosstalk between ferroptosis and other cell death mechanisms?
  - Can the modulation of key regulators involved in the crosstalk between apoptosis, and necroptosis serve as an effective therapeutic strategy for inflammatory diseases?

## Introduction

Cell death is a highly significant biological process essential for achieving homeostasis [1]. On one hand, it occurs naturally to contribute in tissue formation and repair, and on the other hand, it aids in the elimination of any harmful or diseased cells, i.e., pathological cell death [2].

Cell death happens in two ways; programmed cell death (PCD), which is premeditated and orchestrated, happens in response to different signals to achieve growth, maintenance, and the physiological stability of the body. The other way is nonprogrammed or accidental cell death, known as necrosis, typically occurring due to sudden injury or trauma.

PCD is classified into, apoptosis, autophagy, mitophagy, necroptosis, pyroptosis, and ferroptosis. which are explained in detail in the following section and summarized in Table 1. Moreover, their activators and inhibitors are summarized in Table 2.

**Table 1:** The main features of apoptosis, autophagy, mitophagy, necroptosis, pyroptosis, and ferroptosis.

Cell death type	Morphological feature	Biochemical features	Main regulators	Activation signal	Physiological effect	Pathological effect (Diseases)	Ref.
Apoptosis	Enhanced cell membrane permeability, chromatin condensation, pyknosis, karyorrhexis, and cellular atrophy, budding forming apoptotic bodies.	Increasing expression of pro-apoptotic proteins BAX/BAK, suppression of anti-apoptotic proteins BCL-XL, activation of caspases and releasing of cytochrome c.	TNF/Fas, P53, Caspases, BAX, BAK, BCL-XL	Intracellular stimuli, including DNA damage, excessive ROS and increase mitochondrial membrane permeability. Extracellular damaging stimuli or infection by extracellular pathogen resulting in death receptor activation.	Cell death-mediated pathway without inflammation or damaging neighboring cells.	Cancer, bacterial and viral diseases, heart diseases, neurodegenerative, and autoimmune diseases.	[5]
Autophagy	Cytoplasmic vacuolization, phagophore, autophagosome and autolysosome formation, increase lysosomal activity and no chromatin condensation	Formation of ULK1 complex, mTOR pathway inhibition and activation of beclin-1	ATG5, ATG7, LC3-II and beclin-1	Nutrient deprivation, hypoxia and stress	Clearence of damaged cells and nutrients recycling so maintain cell survival, regulate cell life cycle and natural development.	Cancer, neurodegenerative diseases, inflammation, liver diseases, kidney diseases and diabetes mellitus type-II.	[11] [24]
Mitophagy	Formation of autolysosome enclosing the damaged mitochondria	Overexpression of lysosomal enzymes and lysosome activity	PINK1 and parkin BNIP3/NIX	Mitochondrial damage, hypoxia and nutrient deficiency	Terminal differentiation of red blood cells, degradation of paternal mitochondria, and eliminate damaged mitochondria.	Neurodegenerative, skeletal muscle diseases, cardiovascular, and metabolic disorders.	[25, 82]
Necroptosis	Plasma membrane rupture, moderate chromatin condensation, and cytoplasmic swelling	Necrosome complex formation, RIPK1, RIPK3, and MLKL activation followed by phosphorylation, and release of DAMPs	RIPK1/3 and MLKL	Stressors, damaging signals activating death receptors (TNFR)	Minimize cell death.	Cancer, Alzheimer, parkinsonism, multiple sclerosis, pulmonary, liver, enteric, and cardiac diseases	[50]
Pyroptosis	Plasma membrane rupture, cytoplasmic swelling with leakage of cytoplasmic contents, and DNA breaks	Activation of caspase-1 or caspase-4/5/11, GSDMD pore formation and release of inflammatory cytokines	Caspase-1, caspase-4/5/11, GSDMD and IL-18, IL-1β	Cell damage, mitochondrial ROS, DNA breaks, or infection by pathogens	Decrease inflammation.	Atherosclerosis, myocardial infarction, chronic kidney diseases, and ischemia/reperfusion injury	[53]
Ferroptosis	Cell membrane rupture, chromatin condensation, mitochondria atrophy, reduction, sometimes disappearance of mitochondrial ridge	Inhibition of system XC^-^, depletion of GSH, iron-overload, lipid peroxidation, and accumulation of ROS	SLC7A11, GPX4, ACSL4 VDAC2/3 and TFR1	Iron overload, increasing glutamine level, disruption of antioxidant system.	Prevent oxidative stress	Neurodegenerative diseases, autoimmune diseases, chronic obstructive pulmonary disease associated with cigarette smoking, liver, and lung fibrosis, and Pelizaeus–Merzbacher disease a rare genetic disease.	[69, 74]

This table summarizes the distinguishing characteristics of various cell death mechanisms, including their morphological and biochemical features, key regulatory proteins, activating signals, and both physiological and pathological effects.

**Table 2:** Activators and Inhibitors of apoptosis, autophagy, mitophagy, necroptosis, pyroptosis, and, ferroptosis.

Cell death type	Activators	Inhibitors	Ref.
Apoptosis	-FASL, DCC, UNC5B	- Inhibitors of caspases: Z-VAD-FMK, emricasan, Q-VD-OPh - Specific inhibitors; - Inhibitors targeting pancaspase : Z-VAD(OH)-FMK - Inhibitors targeting caspase-3, -6, -7, and -10: Z-DEVD-FMK - Inhibitors targeting caspase-2: Z-VDVAD-FMK - Inhibitors targeting caspase-3: ivachtin - Inhibitors targeting caspase-3 and -7: Ac-DEVD-CHO - Inhibitors targeting caspase-8: Z-IETD-FMK - Inhibitors targeting caspase-9: Q-LEHD-OPh - Activators of apoptosis inhibitors (IAPs) - others: ML-IAP/livin, NAIP.	[14, 64] [240]
Autophagy	Rapamycin, carbamazepine, lithium, sodium, valproate, and C2- ceramide.	- Agents inhibit ULK1: MRT68921, MRT67307, SBI-0206965 - Agents inhibit beclin-1; Spautin-1. - Agents inhibit PI3K: 3-methyladenine, LY294002, wortmannin. - Agents inhibit lysosome: chloroquine, hydrochloroquin. - Agents inhibit H^+^-ATPase: bafilomycin A1, concanamycin A	[14] [240, 241]
Mitophagy	Niclosamide, Toxin T-2, Resveratrol, Urolithin A, Quercetin, Sorafenib, Ketoconazole, Mito-CP and Mito-Metformin, and spermidine.	Liensinine, Haloperidol, and Mdivi-1(Mitochondrial Division Inhibitor 1).	[14, 242, 243]
Necroptosis	PAMPs, Z-VAD-FMK and TNF-α	- RIP1 inhibitor: Necrostatin-1 - RIPK3 inhibitors: GSK872, HS-1371 - MLKL inhibitor: necrosulfonamide - HSP90 Inhibitor: KongensinA - NETs inhibitor: DNase - PADI4: cl-amidine - Others :Tetrahydroisoquinolines, lactoferrin	[14, 145, 240] [244]
Pyroptosis	Metformin, DHA, DPP8/9 inhibitor, anthocyanin L61H10, BI2536, lobaplatin, doxorubicin, α-NETA, cisplatin, paclitaxel, ZnO-NPs, ivermectin, and iron	- NLRP3 inflammasome inhibitors: glybenclamide, MCC950, CY-09, oridonin, and isoliquiritigenin - Caspase- 1 inhibitors: Ac-YVAD-cmk, Z-YVAD (OMe)-FMK, VX765 - Caspase-11 inhibitors: wedelolactone - Inhibitors of GSDMD cleavage: Ac-FLTD-CMK. - Inhibitors of GSDMD: necrosulfonamide, Polyphyllin VI - Others: Disulfiram, LDC7559, Ac-FLTD-CMK, morroniside	[14, 64, 245, 246] [221, 240]
Ferroptosis	GPX4 inactivators; - (class I FINs): erastin and its derivatives, glutamate, cyst(e) inase, sorafenib, buthionine sulfoximine, sulfasalazine, BAY 87–2243, DPI2. - (class II, III FINs): RSL3, FIN56, DPI7/ML162, DPI10/ML210, DPI12, DPI13, DPI17, DPI18, DPI19 Altretamine, withaferin A. Lipid lowering agents; fluvastatin, lovastatin, simvastatin - (class IV FINs): act as iron loaders FINO_2_, hemoglobin, hemin, non-thermal plasma, (NH_4_)2Fe (SO_4_)_2,_ FeCl_2_, lapatinib + siramesine, amino acid depletion +Cornell dots, salinomycin, BAY 11–7085. - Others: Sanguinarine chloride, artemisinin derivatives, CIL41, CIL56, CIL69, CIL70, CIL75, and CIL79, isothiocyanates, gambogic acid, and lanperisone.	- Iron chelating agents: desferoxamine, ciclopirox deferiprone, solamine, and 2, 2- Bipyridyl. Agents inhibits ROS formation: ferrostatin-1, liproxstatin-1, SRS chemical compounds, zileuton β-mercaptoethanol, CoQ10, β-carotene, N- acetyl cysteine, XJB-5-131, and baicalein - Anti-oxidants: vitamin E, Trolox, U0126. - Inhibitors of DPP-4: vildagliptin, alogliptin, and linagliptin - Inhibitors of ASCL4; thiazolidinedione, and rosiglitazone - Activate GPX4; selenium - Others: ebselen, cycloheximide, selenium and aminooxyacetic acid.	[14, 246, 247]

This table summarizes the key activators and inhibitors involved in various cell death mechanisms. Activators are compounds or factors that initiate or promote specific cell death pathways, while inhibitors prevent or reduce cell death by targeting different steps within these pathways.

## Search methodology

Indexed studies on PubMed, Web of Science, and Google Scholar databases were utilized in this review. Articles published since 2017; earlier articles were also considered. The keywords used were as follows: crosstalk, apoptosis, autophagy, ferroptosis, necroptosis, pyroptosis, mitophagy, inducers, inhibitors.

## Classification of cell death

### Non-programmed cell death (necrosis)

A scientific term called “necrosis” was launched, referring to “*nekros*,” a Greek word meaning dead or corpse, and “*osis*,” means process. It is an unplanned cellular death process resulting from an abrupt incident or sudden infection. The underlying cellular mechanism is not well-known exactly, but this process ends with cell swelling and rupture, releasing its contents into the surrounding environment [3]. This explains the occurrence of tissue damage associated with inflammation in the vicinity of the cell, as it activates the immune response against the entire affected area [4]. Notably, necrosis is known as type III cell death.

### Programmed cell death

PCD denotes the regulated mechanism by which cell viability is lost and dies in response to specific signals or stimuli to preserve the stability of the cellular internal environment.

### Apoptosis

“Apoptosis” derived from “*apo*,” means to separate in Greek language, and “*ptosis*,” means “falling off”. The process of apoptosis, known as type I cell death, signifies a deliberate, programmed cell suicide triggered by specific signals received by the cell.

Since, one of the primary differentiating factors among the diverse types of cell death is the cell’s morphology, in apoptosis, the cell undergoes shrinkage and chromatin condensation, followed by the formation of small particles called apoptotic bodies. These bodies are then engulfed and eliminated by immune cells as macrophages, resulting in no inflammation or tissue damage; unlike necrosis, which we discussed earlier. Although apoptosis is considered a pro-survival pathway, it participates in many pathological conditions such as cancer, bacterial and viral diseases, heart diseases, and neurodegenerative and autoimmune diseases [5].

**Fig. 1:**
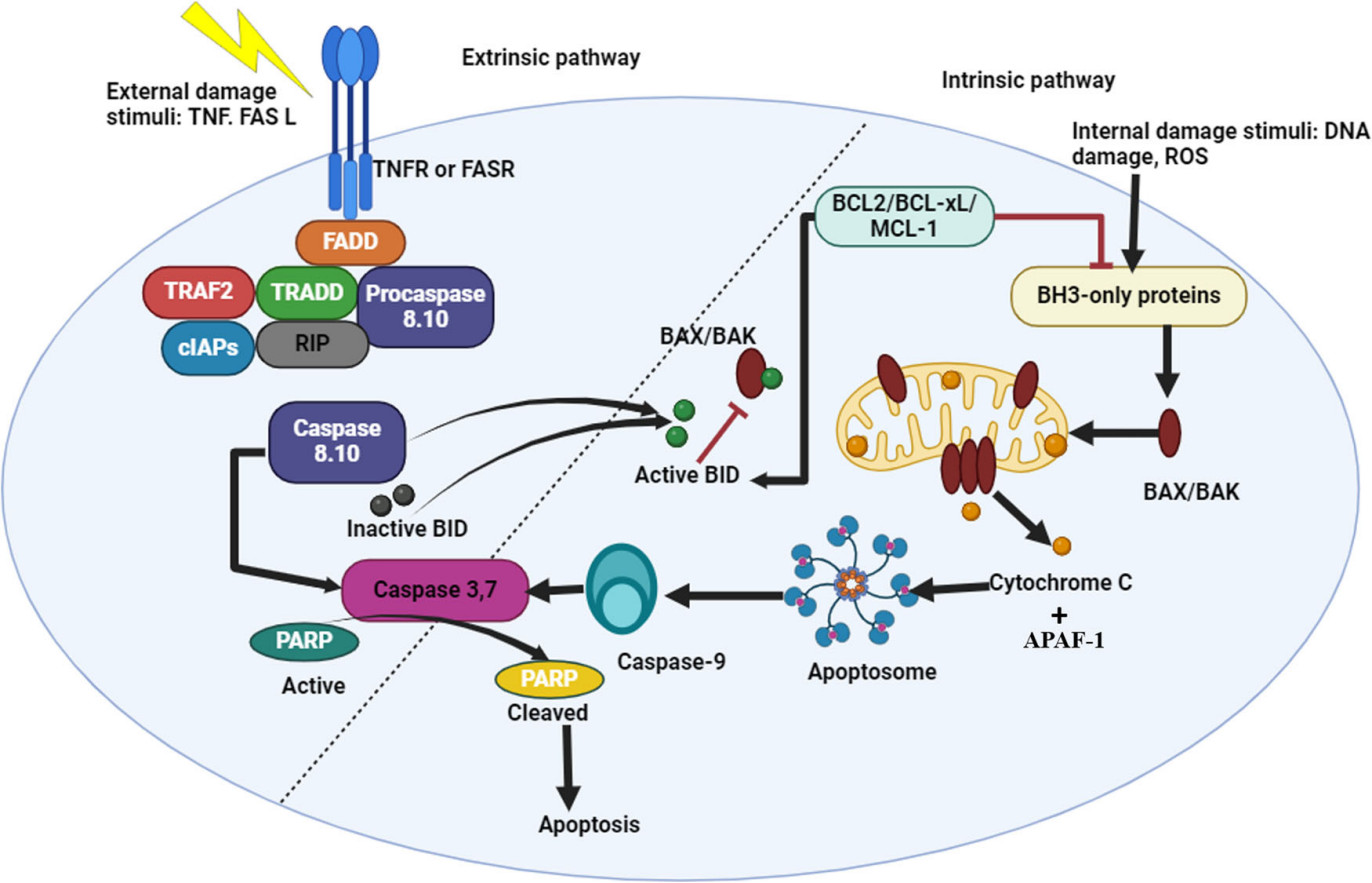
Schematic diagram of pathways to apoptosis. The extrinsic pathway (left side) is initiated by the binding of death ligands, such as FasL or TNF-α, to their respective death receptors on the cell surface, leading to the recruitment of adaptor proteins and the activation of initiator caspase-8. Caspase-8 then activates downstream effector caspases, including caspase-3 and caspase-7, resulting in cell death. The intrinsic pathway (right side) is triggered by intracellular stress signals, such as DNA damage or oxidative stress, which cause mitochondrial outer membrane permeabilization through BAX and BAK activation. This releases cytochrome c into the cytosol, forming the apoptosome complex and activating initiator caspase-9. Both pathways converge on the activation of effector caspases, culminating in apoptotic cell death.

Intrinsic or extrinsic activation pathways are the maestro orchestrating apoptosis induction, determined according to the stimulus that activates the process, as illustrated in Fig. 1.

Activation of the extrinsic apoptosis pathway, named the death receptor pathway, occurs when the external ligands as tumor necrosis factor [TNF]-α or Fas ligand [FasL]) bind to their respective receptors, known as death receptors. This interaction leads to the formation of the death-inducing signaling complex (DISC), which activates caspase-8 and caspase-10, the initiator caspases. Eventually, caspases-3, 6, and 7, named the effector caspases, are activated by the initiator caspases, and the cleaved Poly ADP-ribose polymerase (PARP) is formed, resulting in cell breakdown and triggering the extrinsic apoptosis pathway [6].

Conversely, the intrinsic pathway, named the mitochondrial pathway, is activated by internal cellular disturbances such as oxidative stress, DNA, and/or mitochondrial damage. The intracellular stress, results in disruption in the balance of pro-apoptotic proteins including, Bax family (Bax, Bak, Bok), as well as the BH3- only family (Bid, Bim, Bik, Bad, Bmf, Hrk, Noxa, and PUMA) and the anti-apoptotic proteins, including the Bcl-2 family (Bcl-2, Bcl-xL, Bcl-W, Mcl-1, and A1) [7]. Due to stressors, nuclear P53 becomes activated, subsequently stimulating the p53 upregulated modulator of apoptosis (PUMA), which binds to the anti-apoptotic protein BCL-xl, resulting in its inhibition. Consequently, cytoplasmic P53 retains the ability to activate BAX and BAK, the pro-apoptotic proteins [8]. Bax and Bak results in the opening of the mitochondrial permeability transition pore (MPTP), causing cytochrome c to diffuse into the cytosol. Cytochrome c, along with apoptotic protease activating factor-1 (APAF1), forms the apoptosome, which in turn activates caspase-9, which activates caspase-3, leading to intrinsic apoptotic cell death [6]. Notably, in case of the absence of the anti-apoptotic proteins, the activators, including Bid, Bim, and PUMA can directly bind and activate BAX and BAK [9]. Moreover, sensitizers such as Bad, Noxa, Bik, PUMA can suppress the inhibiting effect of Bcl-2, Bcl-xL, and Mcl-1 on BAX and BAK, thus activating them indirectly [10]. Remarkably, PUMA can act as an activator and a sensitizer.

### Autophagy and autophagic cell death

“Autophagy” is a term that comes from “*auto*,” in Greek means self, and “*phagy*,” means eating or devouring. This process explains a well-organized way by which cells break down and reuse cellular components for various cellular functions. Autophagy is a natural physiological process that occurs in the body to achieve homeostasis by fairly distributing resources and disposing of harmful or non-beneficial materials, thus, regulating cell life cycle and natural development [11]. However, a disruption in this process occurs when cells are subjected to starvation, oxidative stress, or any toxic substance that results in cell death, namely, autophagic cell death or type-II cell death [12]. This dual functionality underscores the pivotal role of autophagy in determining cellular fate, acting as both a survival mechanism and a driver of cell death, depending on the cellular context and external stimuli. It is important to note that, autophagic cell death can be classified into two distinct types. The first is autophagy-dependent cell death (ADCD), including excessive mitophagy, autosis, and endoplasmic reticulum (ER)-phagy, which is directly reliant on autophagy-related molecules and components. This type can be inhibited through genetic silencing or pharmacological blockade of the autophagy process. The second, autophagy-mediated cell death (AMCD), involves the interaction of autophagic machinery with other cell death molecules, or autophagy activates other cell death mechanisms such as apoptosis, ferroptosis, and necroptosis, to facilitate cell demise [13].

Autophagy encompasses three main types: macroautophagy, microautophagy, and chaperone-mediated autophagy, distinguished by their triggering signals, duration of action, target specificity, and mechanisms of transport to lysosomes. In macroautophagy, the material to be degraded is enclosed within a bilayer membrane vesicle, forming an autophagosome, which subsequently fuses with a lysosome for degradation. Microautophagy, unlike macroautophagy, does not involve autophagosome formation; rather, it directly degrades cellular components by engulfing cytoplasmic material at the lysosomal membrane through invagination or septation. Chaperone-mediated autophagy is a selective process where intracellular proteins bind to chaperones for transport to lysosomal compartments, where they undergo enzymatic digestion [14].

The primary driver of the autophagy process is the unc-51-like kinase-1 (ULK1) complex, which is composed of several proteins. The mammalian target of rapamycin (mTOR) pathway is an important pathway that governs the formation or inhibition of this complex. Under normal conditions, mTOR is activated due to its importance in many cellular functions, which prevents ULK1 complex formation. Contrariwise, under certain conditions or starvation, the mTOR pathway is inhibited, leading to ULK1 complex formation [15].

**Fig. 2:**
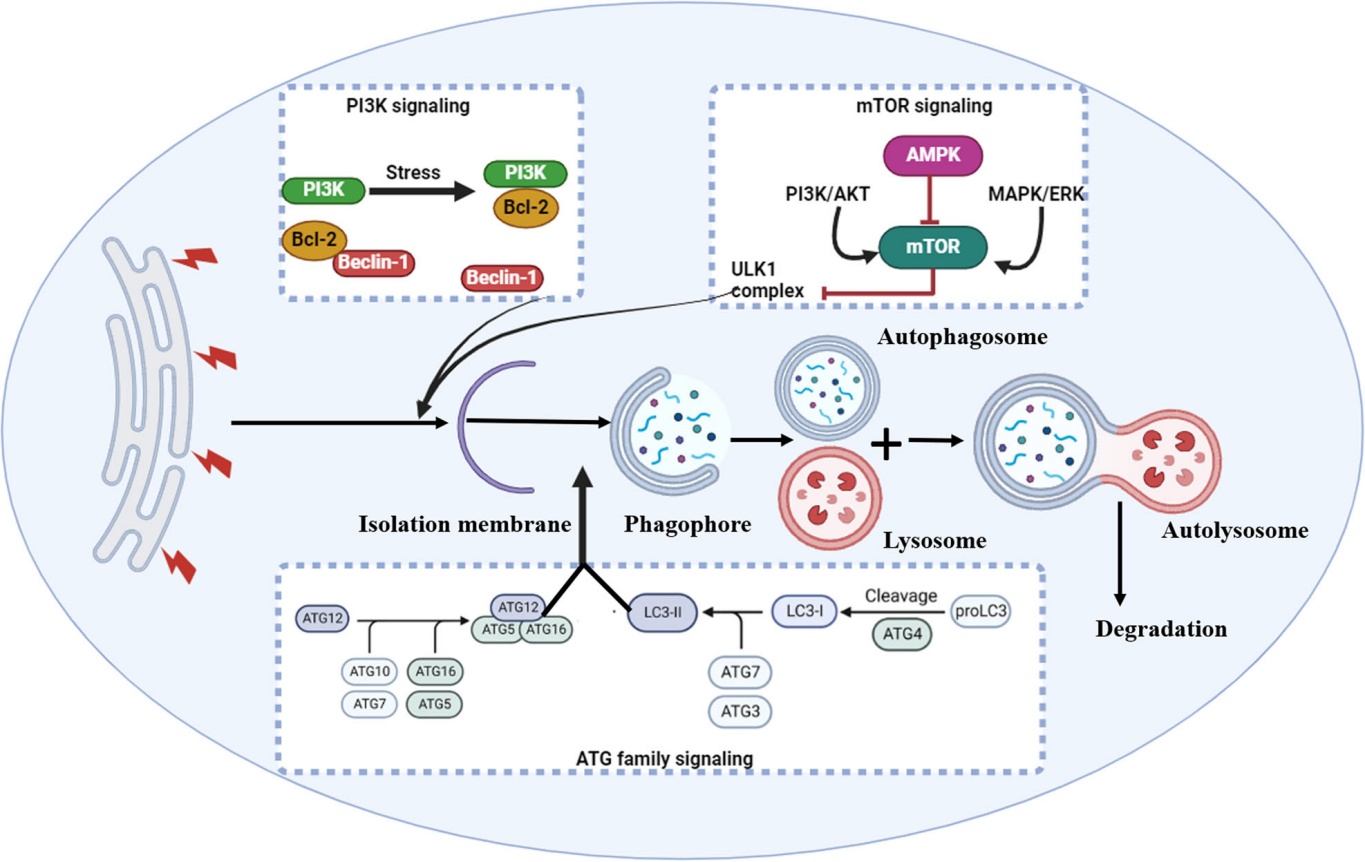
Molecular mechanism of autophagy. Autophagy is initiated by the formation of a double-membrane structure known as the phagophore, which sequesters cytoplasmic components for degradation. This process requires the ULK1 complex, which is regulated by the mTOR pathway. Under nutrient-rich conditions, active mTOR suppresses autophagy by inhibiting the ULK1 complex. However, during stress or nutrient deprivation, mTOR inhibition activates the ULK1 complex, triggering autophagy. The phagophore then expands and captures cytoplasmic components, forming an autophagosome. Autophagosome fuses with lysosomes to form autolysosomes, where degradation occurs. Autophagy is coordinated by various proteins, including the ATG family, Beclin-1, and LC3, which facilitate phagophore formation, elongation, and autophagosome maturation.

This complex initiates the formation of a double-membrane barrier called the phagophore, which elongates and encapsulates the inner cellular components, forming an autophagosome. The autophagosome merges with the lysosome, forming the autolysosome, which digests the cellular components with its enzymes [16], as shown in Fig. 2. Several proteins, including those from the autophagy-related gene (ATG) family and beclin-1, play fundamental roles in the autophagy process by participating in the phagophore formation and recruitment of autophagic proteins [17]. On the other hand, microtubule-associated protein light chain 3 (LC3), is another protein involved in elongating, sealing the phagophore, and maturing the autophagosomes [18]. Indeed, two conjugation systems namely, ATG12 system including ATG12-ATG5-ATG16L covalent bonded complex, and the ATG8 system including ATG3, ATG4 and ATG 7 participate in activation of LC3-I to LC-3II. LC3-II can attach to autophagosome outer membrane, promoting its elongation and maturation, inducing autophagy [19]. On the other hand, PI3k participates in setting beclin-1 free, and autophagy induction under stress conditions [20]. AMPK is a well-known regulator of autophagy processes through phosphorylation and activation of ULK1 during glucose starvation [21]. Notably, P62 is crucial for autophagy completion [22], but its overexpression activates mTOR phosphorylation, suppress expression of beclin-1, LC3-II level, and stops autophagy [23].

Autophagy has been observed in development of many pathological disorders including, neurodegenerative diseases, inflammation, liver, kidney diseases, cancer and diabetes mellitus type-II [24]. Therefore, delving deeper into the study of autophagy is crucial as it promises significant results in the treatment of these diseases.

### Mitophagy

Mitochondria serve as vital organelles regulating cellular energy balance and cell death. Recently, mitophagy (mitochondrial autophagy) has emerged as a pivotal factor in various biological processes, including the terminal differentiation of red blood cells and the degradation of paternal mitochondria. As well as, pathological disorders as neurodegenerative, skeletal muscle diseases, cardiovascular, and metabolic disorders [25].

Mitophagy involves two main stages which are, preparing the damaged mitochondria to be recognized by the autophagic machinery, and attraction of the autophagic proteins. Current advancements in mitophagy research highlight the involvement of either the PTEN-induced kinase-1 (PINK1)/Parkin signaling pathway or the outer mitochondrial membrane receptors NIP3-like protein X (NIX) and B‐cell lymphoma 2 (BCL-2)/adenovirus E1B 19‐kDa‐interacting protein 3 (BNIP3) in priming damaged mitochondria for removal and inducing mitophagy [26].

PINK1 is a serine-threonine kinase, while Parkin is an E3 ubiquitin ligase. In normal circumstances, PINK1 enters the mitochondria and cleaved by presenilin-associated Rhomboid-like, inner membrane protease, then undergoes cytosolic proteosome degradation. However, in the case of depolarized mitochondria, the outer membrane of mitochondria becomes crowded with PINK1, instead of being imported and cleaved [27]. Notably, the phosphoglycerate mutase family member 5 stabilizes PINK1 on damaged mitochondrial membrane, and has the ability to attach to LC3 proteins [28]. Subsequently, PINK1 phosphorylates mitofusin1 (MFN1), an outer mitochondrial membrane (OMM) protein, as well as activates and recruits Parkin [29, 30]. Parkin functions by ubiquitinating voltage-dependent anion channel (VDAC), MFN1, and mitochondrial Rho GTPase proteins [31]. The formed ubiquitinated proteins serve as excellent ligands for autophagic receptors, facilitating mitophagy. Interestingly, PINK1, has the ability to recruit autophagic receptors in a parkin-independent way, including nuclear dot protein 52 kDa (NDP52) and optineurin (OPTN) [32]. NDP52 facilitates the recruitment and activation of ULK1 on cargo through its association with the NDP52-FIP200/ULK1 complex [33]. Furthermore, OPTN interacts not only with ATG8 proteins but also with ATG9A molecules, activating mitophagy and autophagy [34, 35].

**Fig. 3:**
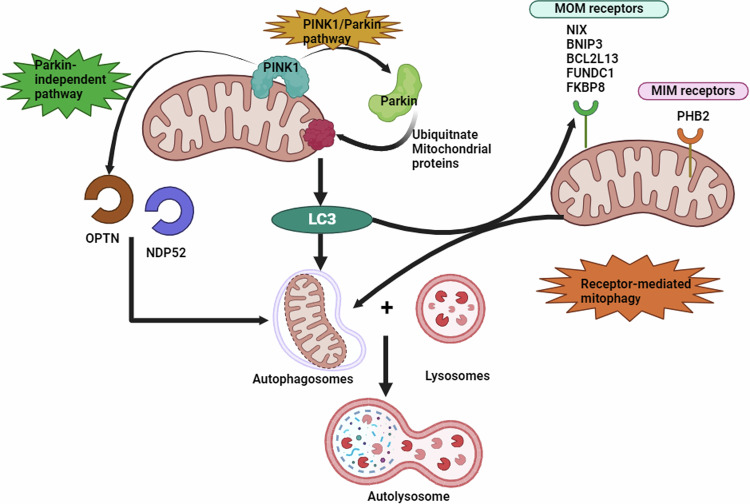
Summary of the main mitophagy pathways. Mitophagy is primarily controlled through two main pathways. The first involves the PINK1/Parkin signaling pathway, where damaged mitochondria retain PINK1 on their surface, leading to the recruitment and activation of Parkin. Parkin ubiquitinates specific mitochondrial proteins, creating signals that attract autophagy receptors such as NDP52 and OPTN, which bind to autophagy machinery to drive mitophagy. The second pathway includes mitochondrial receptors like BNIP3 and NIX, which, when phosphorylated, bind directly to LC3/GABARAP proteins on the autophagosome membrane, facilitating mitochondrial degradation. Other receptors, including FUNDC1, BCL2L13, FKBP8, and PHB2, further regulate mitophagy by modulating these processes and enhancing mitochondrial clearance.

BNIP3/ BNIP3L(NIX) is another alternative pathway that mediates mitophagy, besides participating in apoptosis and necroptosis mediating cell death [36, 37]. BNIP3 and NIX are receptors found normally on the OMM, in an inactive form. However, upon mitophagy induction, the Ser212‐ phosphorylated BNIP3/NIX monomers, activated and dephosphorylated at the c-terminus of those receptors. More stable BNIP3/NIX (Ser34, and Ser35) phosphorylated dimer is formed instead, which directly binds to Atg8 homologs, and the LC3/GABA type A receptor‐associated protein (GABARAP) proteins (LC3/GABARAP). Those proteins are found on the autophagosome membrane, and attach to BNIP3/NIX through a conservative LC3‐interacting region (LIR) domain, mediating mitophagy [38]. Notably, other receptors were found on the OMM linked to activation of mitophagy, including BCL-2 like 13 (BCL2L13), FUN14 domain containing 1 (FUNDC1), autophagy and beclin-1 regulator 1 (AMBRA1), and FKBP prolyl isomerase 8 (FKBP8), and recently, inner mitochondrial membrane receptor, prohibitin 2 (PHB2) [39]. It is worth to mention, that the regulatory proteins sirtuins, including SIRT1, and SIRT3 are PINK1/Parkin pathway upstream activators [40, 41]. Sirtuins, can mediate mitophagy directly by affecting post-translational modification of mitophagy proteins as ATG 5, ATG7, and ATG8, or, indirectly by increasing expression of mTORC1, PARK1, beclin-1, BNIP3 [42], as demonstrated in Fig. 3. Notably, dynamin-related protein 1 (Drp1), a GTP-binding protein, that plays a significant role in mitochondrial fission. Drp1, originally found in the cytosol, however it translocated to the mitochondria to allow damaged mitochondria segregation and mitophagy [43]. Parkin, also regulates Drp1 activity and degradation by polyubiquitination [44].

### Necroptosis

Necroptosis is the regulated form of necrosis, sharing some characteristics of both necrosis and apoptosis but differs in others. Necroptosis happens as a result of interaction between the ligands and their death receptors, including, Fas receptor, TNF receptor 1 (TNFR1), IFN receptors (IFNRs), toll- like receptors (TLRs), or by DNA-sensing molecules, including DNA-dependent activator of IFN-regulatory factors (DAI), known also as Z-DNA-binding protein 1 (ZBP1) [45]. The TNFR1-dependent necroptosis pathway involves TNF-α binding to TNFR1 and recruiting a series of proteins, forming DISC complex, known as complex I. This complex includes, TNFR-associated death domain (TRADD), receptor-interacting protein kinase-1 (RIPK1), TNFR-associated factor 2 (TRAF2), linear ubiquitin chain assembly complex (LUBAC), cellular inhibitor of apoptosis proteins (cIAP1), cylindromatosis (CYLD), and NF-κB essential modulator (NEMO). The first choice is avoiding cell death and maintain cell survival, where RIPK1 is polyubiquinated by cIAp1 or LUBAC, followed by recruitment of the transforming growth factor-*β*-activated kinase-1 (TAK1) and its adaptor NEMO towards RIPK1. Subsequently, NEMO (also called IKK*γ*) recruits IKKα/IKKβ, forming the inhibitor of I-κB kinase complex (IKK). IKK complex phosphorylates and degrades IkB, a NF-κB inhibitor, consequently activating NF-κB signaling pathways and cell survive [46].

**Fig. 4:**
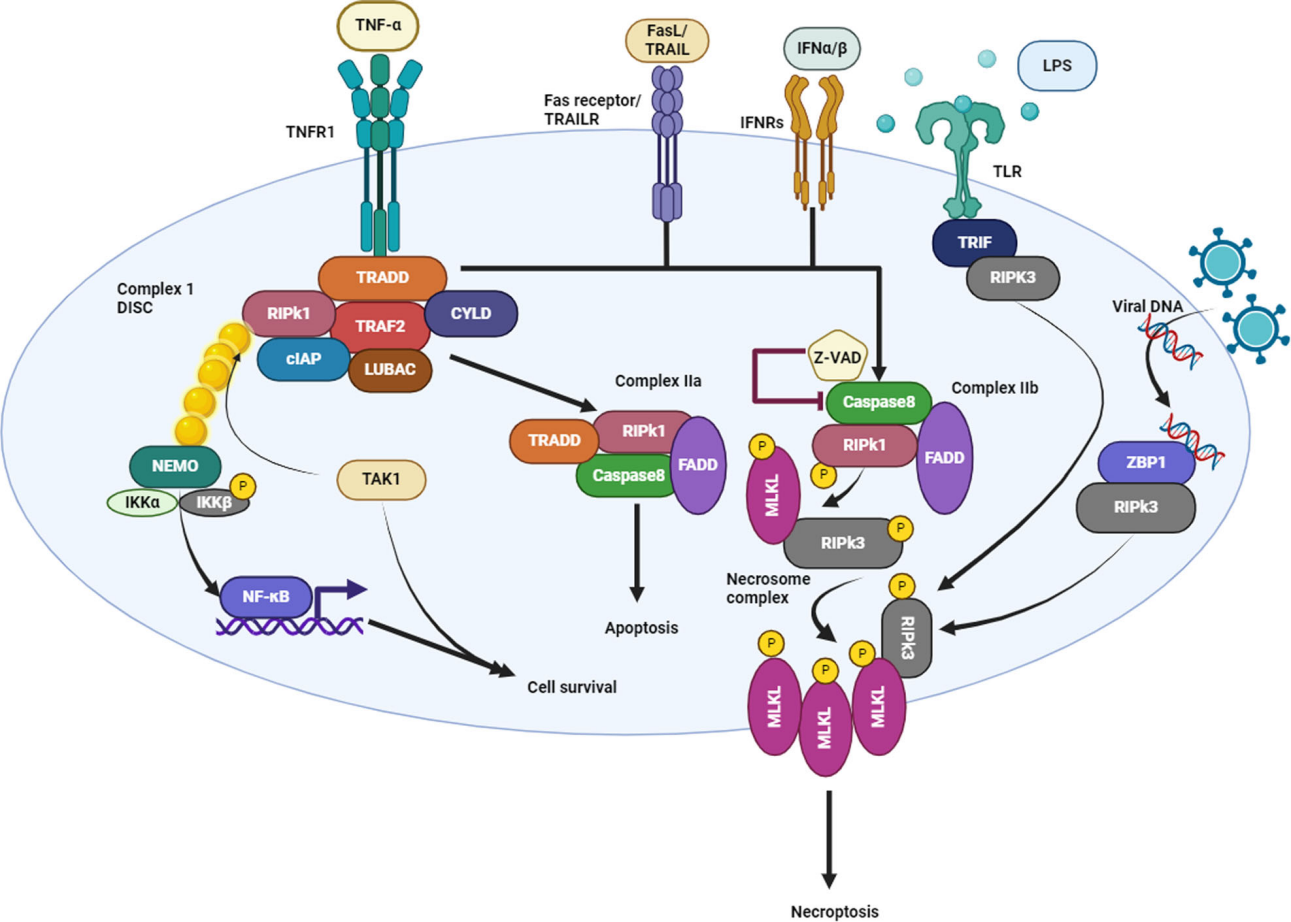
Schematic diagram of pathways involved in necroptosis. The necroptosis pathway is initiated by the interaction between ligands and their death receptors, such as Fas, TNFR1, IFNRs, TLRs, or DNA-sensing molecules like ZBP1/DAI. In the TNFR1-dependent pathway, TNF-α binds to TNFR1, forming complex I (DISC) where RIPK1 is polyubiquitinated by cIAP1 or LUBAC, activating NF-κB signaling to promote cell survival. When NF-κB activation is suppressed, RIPK1, FADD, and caspase-8 form complex IIa, inducing apoptosis. If caspase-8 is inhibited or suppressed, RIPK1, FADD, and caspase-8 form complex IIb, leading to RIPK3 and MLKL activation, which forms the necrosome. Phosphorylated MLKL translocates to the plasma membrane, forming pores, resulting in membrane rupture and necroptosis.

In instances when NF-κB activation is suppressed, deubiquitinated RIPK1, Fas-associated protein with death domain (FADD), TRADD, and pro-caspase-8 assemble as complex IIa (ripoptosome). Complex IIa induces apoptosis through activated caspase-8 and RIPK1 cleavage, which is the second choice [47]. In the case of inhibition of cIAp1, complex IIb is formed, which consists of RIPK1, FADD, and caspase-8. In this case, while TRADD is absent, cells can still undergo apoptosis, where caspase-8 is activated by RIPK1. However, in the absence or inhibition of caspase-8, cells reach the third choice, where RIPK1 recruits and activates receptor-interacting protein kinases 3 (RIPK3) and mixed lineage kinase domain-like protein (MLKL), forming, necrosome complex [48, 49]. Cytosolic phosphorylated MLKL translocate to the plasma membrane, oligomerizes and forms pores, resulting in cell membrane rupture, necroptotic cell death and release of danger-associated molecular patterns (DAMPs). PGAM5, acts as an anchor to necrosome complex, facilitating pores formation. Collectively, we conclude that TNFR1 signaling diverges into distinct complexes, each mediating specific cellular fates. Complex I activate NF-κB, supporting cell survival and inflammatory responses. Complex IIa induces apoptosis via caspase-8, independent of RIPK1, whereas complex IIb initiates caspase-8-dependent apoptosis that requires RIPK1 kinase activity. In cases where caspase-8 is absent or inhibited, necrosome formation is triggered, leading to MLKL-driven necroptosis, as represented in Fig. 4. In necroptotic cell death, immune cells recognize DAMPs, triggering inflammation to eliminate harmful cells and repair damaged ones. If the inflammatory pathway remains uncontrolled, it can lead to prolonged and unwanted inflammation, causing diseases as cancer, Alzheimer, parkinsonism, multiple sclerosis, pulmonary, liver, enteric, and cardiac diseases [50]. Notably, another alternative interaction, including, TLR3/TLR4 activation by dsRNA or LPS, or detection of viral DNA by DAI, results in the formation of a TRIF-RIPK3 complex or ZBP1/DAI-RIPK3 complex, leading to necrosome formation independently of RIPK1. TRIF is the Toll/IL-1 receptor domain-containing adapter- inducing INF-β [51].

### Pyroptosis

Pyroptosis represents an orchestrated process of inflammatory PCD, which was initially observed by Zychlinsky et al. in macrophages infected with Shigella Flexneri [52]. Although pyroptosis serves as an innate immune response against infections and contributes to the normal functioning of the immune system, but, excessive pyroptosis can provoke immune dysregulation in numerous pathological conditions including, atherosclerosis, myocardial infarction, chronic kidney diseases, and Ischemia/reperfusion injury [53].

Pyroptosis is primarily categorized into two main pathways; canonical pathway, which involve caspase-1, and non-canonical pathways, which rely on caspase-11 in mice and caspase-4 and/or caspase-5 in human cells. Eventually, results in various gasdermin family members cleavage. Cleaved gasdermins participates in membrane perforation and rupture, inflammatory mediators’ secretion, and cell death.

The canonical pathway of pyroptosis necessitates the engagement of inflammasomes, which are large proteinaceous complexes formed in response to various external stimuli such as hypoxia, injury, and pathogens. A typical inflammasome complex comprises a pattern recognition receptor (PRR), a downstream adaptor-like apoptosis-associated speck-like (ASC) protein, and pro-caspase-1. Four primary inflammasome receptors have been recognized: NLR members NOD-, LRR-, and pyrin domain containing 1 (NLRP1); pyrin domain containing 3 (NLRP3); NOD-, LRR-, and caspase recruitment domain containing 4 (NLRC4); and absent in melanoma 2 (AIM2), where they serve as a sensor for different activating signals.

Canonical inflammasome is prepared in two subsequent steps: priming signal, then assembly and activation signal. Firstly, the priming signal, upon exposure to pathogen-associated molecular patterns (PAMPs) or DAMPs, the PRR, including TLRs or TNFR, undergo phosphorylation. Subsequently, the transcription of nuclear factor kappa B (NF-κB) is activated. Within the nucleus, NF-κB stimulates the transcription and the translation of NLRP3, pro-IL-1β, and pro-IL-18, which remain inactive in the cytoplasm [54]. Secondly, a subsequent stimulus, including an increase in ROS, PAMPs, DAMPs, autophagic dysfunction, mitochondrial damage, and lysosomal damage, triggers the assembly and activation signal. Under the action of such stimuli, the oligomerization and assembly of the inactive NLRP3, ASC, and pro-caspase-1 are activated, forming inflammasome complex. The inflammasome complex facilitates the activation of pro-caspase-1 into caspase-1, which has two main roles: cleavage of immature pro-interleukins into their mature forms IL-1β and IL-18. Moreover, caspase-1 cleaves gasdermin-D (GSDMD) into N- and C-terminal fragments. The N-terminal domains of GSDMD bind to cell membranes, forming oligomeric pores. Cell swelling, plasma membrane rupture, and release of intracellular content are a classical consequence for the perforated cell, finally resulting in cell death [55]. Notably, gasdermins, the pyroptosis executor, are divided into six species in humans, including, GSDM A to E and an additional DFNB59, where they participate in many mechanisms mediating cell death [56].

**Fig. 5:**
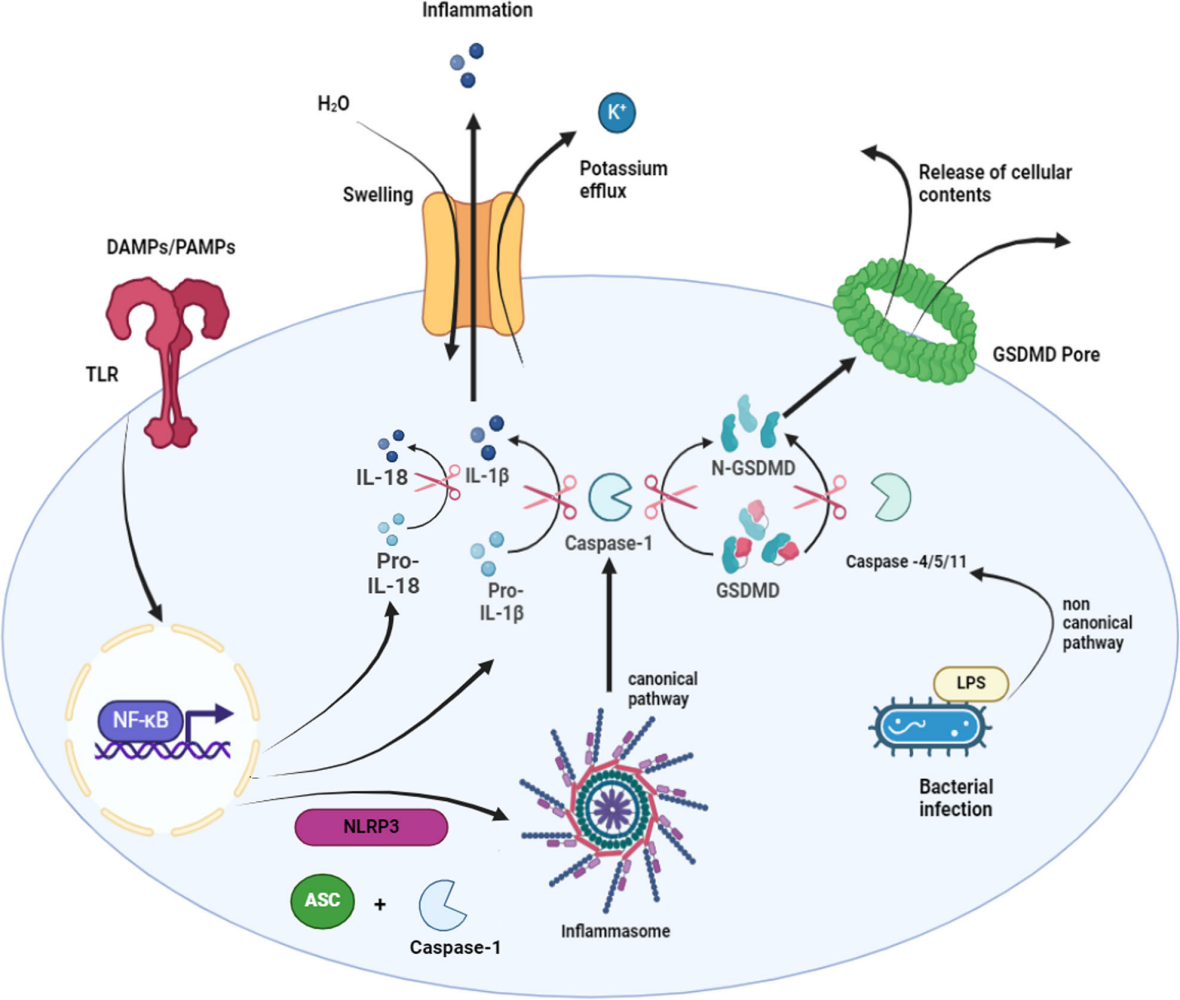
Mechanisms of pyroptosis including canonical and noncanonical pathways. The canonical pyroptosis pathway is initiated by the activation of TLRs or TNFRs in response to DAMPs or PAMPs, leading to NF-κB activation and transcription of pro-inflammatory cytokines (pro-IL-1β and pro-IL-18) and NLRP3. Following a second stimulus, such as ROS, mitochondrial or lysosomal damage, NLRP3 inflammasomes assemble, activating caspase-1. Caspase-1 cleaves pro-IL-1β and pro-IL-18 into their mature forms and activates gasdermin-D (GSDMD). The N-terminal fragment of GSDMD oligomerizes and forms pores in the plasma membrane, ultimately leading to pyroptotic cell death. In the non-canonical pathway, bacterial lipopolysaccharides (LPS) bind directly to caspase-4/5 (in humans) or caspase-11 (in mice), activating them and triggering GSDMD cleavage and pore formation.

In the non-canonical pathway, lipopolysaccharides (LPS) loaded in vesicles from gram-negative bacteria enter the infected cell. Inside, the LPS bind to caspase- 4/5 in humans and, similarly, bind to caspase-11 in mice, activating them. The complex originated is called the non-inflammasome complex, which in turn activates GSDMD and forms membrane pores, enabling pyroptotic cell death [55]. Since pyroptosis is an inflammatory cell death process, active inflammatory cytokines must be released to complete the non-canonical pathway. Recent studies have revealed that caspase-4/5/11 acts both as receptors and effector molecules in the non-canonical pathway [57]. Caspase-4/5/11 has the ability to open Pannexin-1 channels, allowing K^+^efflux, thereby activating NLRP3 inflammasome and completing interleukin maturation [58]. Another activation mechanism involves the previously formed active GSDMD, which can indirectly activate caspase-1, continuing the process in a way similar to the canonical pathway until interleukins are matured and activated [59], as illustrated in Fig. 5.

### Ferroptosis

Ferroptosis is a Fe-dependent form of PCD, hence its name. Ferroptosis is regulated by three main pathways, highlighting iron metabolism, lipid peroxidation, and antioxidant systems [60]. Iron is essential for the body and obtained by the cells through intestinal absorption and erythrocyte breakdown. Transferrin protein (TF) binds iron, forming a complex, which is then internalized into the cell via transferrin receptors (TFR1), by endocytosis. Fe^3+^ enters the endosome through the six-transmembrane epithelial antigen of the prostate 3 (STEAP3) and is reduced to Fe^2+^, then released into the cytoplasm via divalent metal transporter 1 (DMT1). Unused iron can be stored either in the mitochondria or cytoplasm as a labile iron pool (LIP) or stored in the form of ferritin with the assistance of chaperones [61]. Notably, ferroportin (FPN), is the only protein responsible for exporting iron from intracellular to extracellular compartment, having an important role in ferroptosis [62]. Under pathological conditions, dysregulation in genes controlling iron metabolism including, TF, TFR1, FPN, DMT1 will cause a substantial elevation in intracellular iron levels [63, 64]. Also, ferritinophagy mediated by nuclear receptor coactivator- 4 (NCOA4) involving ferritin degradation, activates ferroptosis by increasing intracellular iron content [65].

**Fig. 6:**
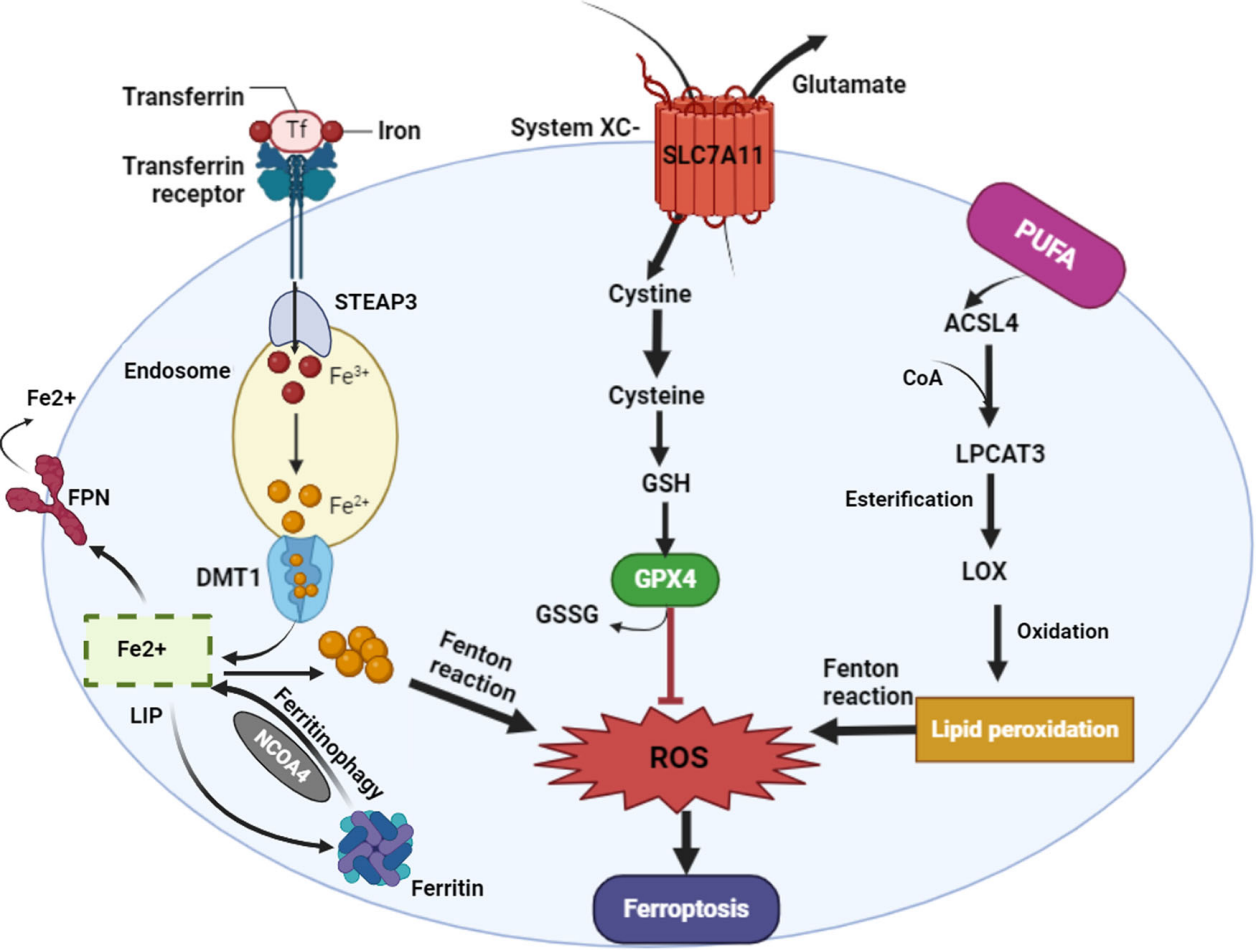
Schematic illustration of the occurrence of ferroptosis. Iron enters cells by binding to transferrin receptor 1 (TFR1) on the cell membrane. Inside the cell, STEAP3 reduces ferric iron (Fe3+) to ferrous iron (Fe2+), which is then released into the cytoplasm via divalent metal transporter 1 (DMT1) and stored in the labile iron pool or ferritin. Ferritinophagy, mediated by nuclear receptor coactivator-4 (NCOA4), promotes the degradation of ferritin, increasing free iron levels. Free iron generates reactive oxygen species (ROS) through the Fenton reaction, leading to lipid peroxidation through the activation of lipoxygenases (LOX) and other enzymes. GPX4, the primary defense against lipid peroxidation relies on glutathione (GSH) as a cofactor. System Xc−, normally imports cystine in exchange for intracellular glutamate. Cystine is converted to cysteine, a precursor for GSH synthesis. When the cystine/glutamate antiporter SLC7A11 is inhibited, intracellular cysteine levels drop, impairing GSH production and GPX4 activity. This depletion of GPX4 and GSH hampers ROS and lipid peroxide neutralization, triggering oxidative stress and ferroptotic cell death.

Additionally, lipid peroxidation extensively happens, where lipoxygenases (LOX), lysophosphatidylcholine acyltransferase 3 (LPCAT3), and acyl-CoA synthetase long-chain family member 4 (ACSL4) enzymes become overly active, leading to polyunsaturated fatty acids (PUFA) oxidation, and accumulation of lipid peroxides. PUFA are ordinary components of the cell membrane, having many biological functions. Taking arachidonic acid as an example of PUFA, it undergoes CoA ligation mediated by ASCL4, followed by esterification by LPCAT3 into phosphatidylethanolamine. Subsequently, the arachidonic acid-phosphatidylethanolamines undergo enzymatic oxidation by LOX or non-enzymatically by autoxidation, accumulating lipid peroxides. Eventually, lipid peroxides interact together with intracellular iron overload causing fenton reaction, resulting in increased ROS levels, cell membrane damage, and death [63], as displayed in Fig. 6. In addition to the role of iron in fenton reaction, iron-containing compounds as heme and Fe-S cluster are important activators for many oxidizing enzymes as LOX and cytochrome P450 [66].

Regarding the antioxidant system, such as glutathione (GSH), in dealing with the overproduced reactive oxygen species (ROS), the third blow comes into play. In ferroptosis, active GSH decrease due to the inhibition of Xc- system including, solute carrier family 7 member 11 (SLC7A11). SLC7A11 is a cystine/glutamate antiporter, cystine is reduced to cysteine inside the cells by GSH or thioredoxin reductase 1 (TXNRD1) [67]. Hence, the antiporter inhibition results in depleting intracellular cysteine levels; a glutathione precursor [68, 69]. This occurs in parallel with the increase in the intracellular glutamate levels, which is converted into glutamine by glutaminase enzyme, taking part in tricarboxylic acid cycle (TCA) and ROS production [70]. Moreover, a significant decrease in the activity of glutathione peroxidase 4 (GPX4), a GSH-utilizing antioxidant enzyme, so the ROS and oxidative stress increases [71]. Eventually, the ROS and peroxides cannot be neutralized adequately, inducing ferroptotic cell death and releasing of DAMPs. The tumor-suppressor gene p53 plays a role in this pathway, by inhibiting SLC7A11 antiporter, and activating ferroptosis [72]. Also, using erastin and RSL3 as ferroptosis inducers represents a new cancer treatment approach, by blocking Xc- system or inhibiting GPX4 expression, respectively [73]. Therefore, more research is needed to trace this pathway and discover new agents for its regulation for the management of various diseases including, neurodegenerative diseases, autoimmune diseases, chronic obstructive pulmonary disease associated with cigarette smoking, liver, and lung fibrosis, and the rare genetic disease Pelizaeus–Merzbacher [74].

### Crosstalk and interconnection among necroptosis, pyroptosis, ferroptosis, autophagy, mitophagy, and, apoptosis

**Fig. 7:**
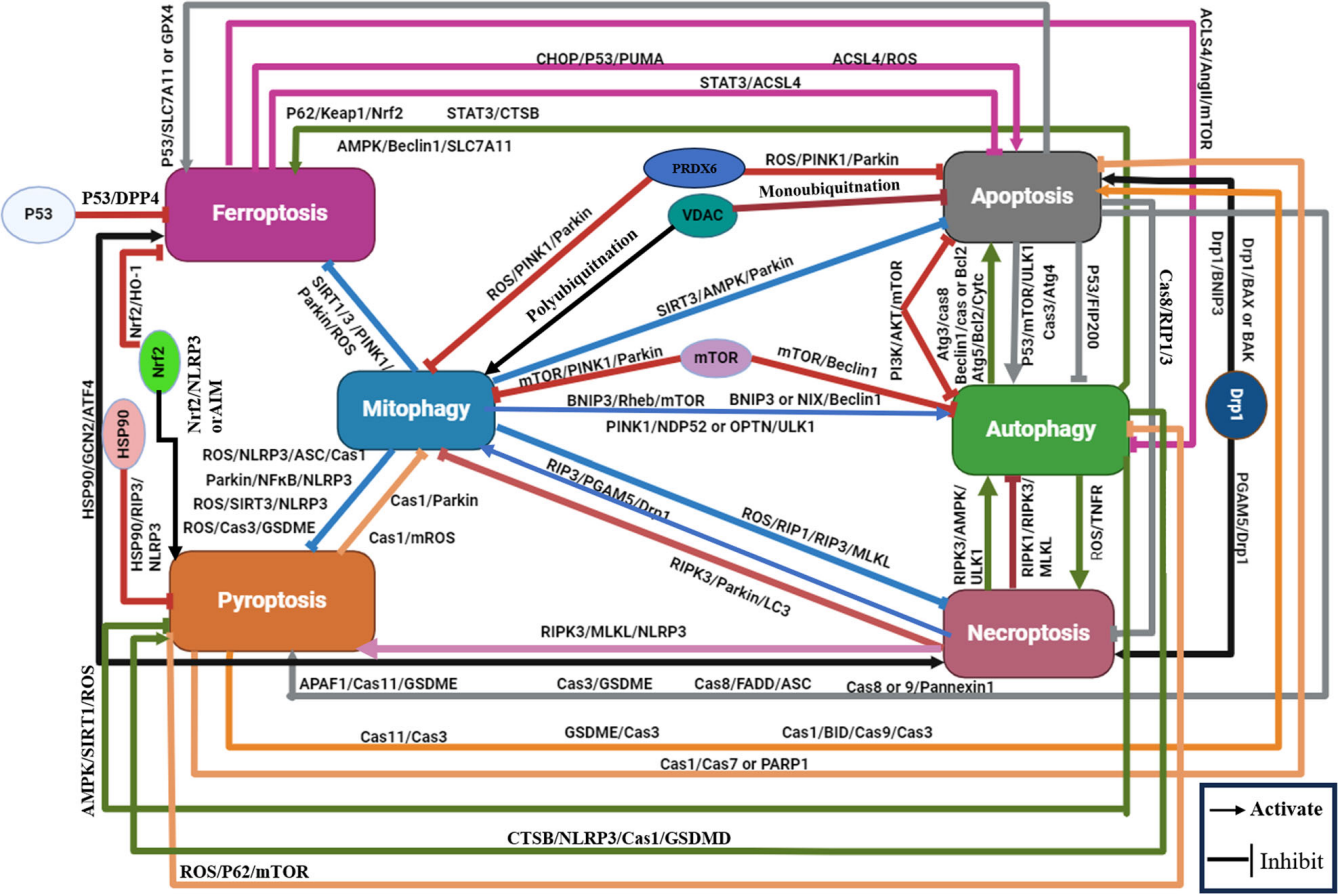
Summary of the crosstalk mechanisms between apoptosis, autophagy, mitophagy, pyroptosis, necroptosis, and ferroptosis. Where each pathway displayed in a colored block emits arrows of the same color directed toward other blocks of other cell death pathways. These arrows indicate a positive or negative influence through the molecular mechanism mentioned on the arrow. Alternatively, red arrows emitted from one block to another, indicating an inhibitory effect through the mechanisms labeled on the arrows. Black arrows refer to a common pathway or specific marker that positively affects both pathways.

Examining various pathways contributing to cell death and assessing their interplay in pathophysiological processes, would enhance our comprehension of the mechanisms regulating PCD and their significance in intricate diseases, as summarized and illustrated in Fig. 7, where each pathway displayed in a colored block emits arrows of the same color directed towards other blocks of other cell death pathways. These arrows indicate a positive or negative influence through the molecular mechanism mentioned on the arrow. Alternatively, red arrows emitted from one block to another, indicating an inhibitory effect through the mechanisms labeled on the arrows. Black arrows refer to a common pathway or specific marker that positively affects both pathways.

### Mitophagy and pyroptosis

The crosstalk between mitophagy and pyroptosis, is attributed to the role of mitophagy as an ROS scavenger. Mitophagy has the ability to eliminate damaged mitochondria that are supposed to generate excessive mitochondrial reactive oxygen species (mtROS), thereby reducing its levels. This affects the NLRP3/ASC/caspase-1 pathway, as mtROS has the capability to activate NLRP3 and form the inflammasome complex [75]. Quercetin has been demonstrated to inhibit pyroptosis-related proteins by promoting mitophagy, thereby alleviating neuronal injury [76]. Similarly, in an invitro hypoxic-ischemic brain damage model, mesenchymal stem cell-derived exosomes enhanced mitophagy, resulting in the suppression of pyroptosis and the protection of microglial cells. Thus, the inhibition of mitophagy was found to trigger pyroptosis [77]. In a recent study, PINK1-mediated mitophagy plays a protective role in postoperative cognitive dysfunction by suppressing caspase-3/GSDME-dependent pyroptosis [78]. Furthermore, SIRT3, one of the key regulators of mitophagy, decreases the activation of the NLRP3 inflammasome, in human macrophages, by reducing ROS [79]. Another key regulator of mitophagy is parkin, which has the ability to inhibit NF-κB by upregulating the expression of protein 20 (A20), anti-apoptotic protein, thus inhibiting inflammasome activation [80]. Consequently, mitophagy can attenuate pyroptosis. On another note, pyroptosis has the ability to inhibit mitophagy through the blockade of its pathway, primarily orchestrated by caspase-1 which cleaves parkin, a key component for completing the mitophagy pathway, halting the mitophagy pathway [81, 82]. Additionally, Caspase-1 enhances mtROS production, mitochondrial permeability, and fragmentation, rendering the damage severe beyond mitophagy repair capacity [81]. Surprisingly, a recent study showed that in rat lung cells, ozone exposure increases cellular ROS levels, which activate PINK1-Parkin-mediated mitophagy and subsequently promote NLRP3 inflammasome-mediated pyroptosis [83]. This finding challenges the previously held belief that mitophagy and pyroptosis inhibit each other, suggesting that other alternative pathways may link these processes and disrupt the conventional feedback mechanisms between them.

### Mitophagy and ferroptosis

Mitophagy plays a role in regulating ferroptosis through two influential factors: iron overload and ROS. Despite, the fact that ROS stimulate both mitophagy and ferroptosis pathways, as mentioned earlier, mitophagy eliminates excess mtROS by disposing of dysfunctional mitochondria, thus reducing ferroptosis [84]. This is the primary mechanism by which mitophagy mitigates ferroptosis. However, there are some controversial studies showing that mitophagy can positively regulate ferroptosis by increasing cellular ROS. For instance, inhibition of mitochondrial complex I by BAY 87–2243 in melanoma cells activates PINK1-dependent mitophagy. This process increases cellular ROS levels, leading to the depletion of GSH and activating lipid peroxidation, promoting ferroptosis [85]. Similarly, another recent study stated that mitophagy-mediated ferroptosis by increasing cellular ROS [86]. Additionally, mitophagy regulates the mechanism of iron translocation between cytoplasm and mitochondria, so that iron is used in many biological processes as heme synthesis and Fe-S cluster synthesis, which is essential in enzyme activity, DNA synthesis, and repair [87]. Hence, mitophagy can contribute to ferroptosis by facilitating the release of ferrous ions through the degradation of Fe-S clusters within the mitochondria [88]. However, Mitoferrins, Mfrn1 and Mfrn2, which are crucial gateways for iron passage through the inner mitochondrial membrane, are disrupted by mitophagy, preventing excessive iron release from mitochondrial matrix to the cytoplasm and, consequently, reducing ferroptosis [89]. Furthermore, VDAC, one of the key regulators of the OMM permeability, plays a role in mitophagy and also, involved in controlling the passage of metabolites, ions, and other molecules between the mitochondria and the cytoplasm. VDAC’s role in activating ferroptosis is primarily linked to its influence on cellular iron homeostasis, ROS generation, and mitochondrial dysfunction [90].

Interestingly, a study revealed that, as a result of abnormalities in expression of SIRT1, and SIRT3 in myocardial ischemia-reperfusion injury model, PINK1/Parkin pathway is inhibited, inhibiting mitophagy, increasing ROS, and activating ferroptosis [91]. Moreover, the insufficiency of FUNDC1, a mitophagy receptor protein, was found to activate ferroptosis in high-fat diet mice, through modulation of ASCL4 [92]. Nonetheless, a recent study has demonstrated that upregulated FUNDC1 in fibrotic liver tissues, facilitates the translocation of GPX4 from the cytoplasm to the mitochondria, where it is degraded along with damaged mitochondria via mitophagy. This process results in the loss of its antioxidant function, thereby promoting the ferroptosis pathway [93]. This underscores the dual role of FUNDC1 in modulating ferroptosis, with its impact differing based on the specific disease context and underlying mechanisms. Protein O-GlcNAcylation, a process that acts as a sensor for glucose flux and regulation of ferritin uptake, plays a role in the interconnection between ferroptosis and mitophagy. Upon pharmacological or genetic inhibition of O-GlcNAc transferase, ferritinophagy (NCOA4-mediated autophagy) and mitophagy are activated. Consequently, more labile iron accumulated, more ROS production, which results in increasing sensitivity towards ferroptosis [94].

A study conducted by Zukor et al. demonstrated that heme oxygenase-1 (HO-1), an iron degradation enzyme, induces mitochondrial damage and autophagy, disposing redox-active iron, consequently reducing ferroptosis [95]. However, in another scenario, HO-1, catalyzes the breaking down of heme, iron production, down-regulate GPX4, inducing lipid peroxidation and ferroptosis [96]. In cisplatin-induced acute kidney injury, BNIP3 and PINK1-Parkin-mediated mitophagy mitigate the excessive release of ROS and the expression of HO-1, thereby inhibiting lipid peroxidation and ferroptosis through the ROS/HO-1/GPX4 axis [97]. These controversial results were attributed to the severity of damage and duration of HO-1 overexpression. In mild damage, HO-1 expression stimulates mitophagy and the removal of damaged mitochondria. While lately, iron accumulates as a result of HO-1 overexpression, activating ferroptosis [98]. So, we can conclude that mitophagy may inhibit or activate ferroptosis, depending on the context, level of cellular and mtROS, and the involved mechanism.

### Mitophagy and necroptosis

Mitophagy has the potential to regulate necroptosis by modulating ROS as well, encompassing O2-derived free radicals and non-radical species [99]. The intracellular ROS can instigate the opening of the MPTP, thereby contributing to RIP1/RIP3/MLKL pathway, inducing necroptosis [100]. Similarly, ROS originating from mitochondria promotes autophosphorylation of RIP1, facilitating the recruitment of RIP3 to form the necrosome complex (RIP1/RIP3), instigating TNF-α-mediated necroptosis [101]. Activation of PINK1-Parkin-mediated mitophagy in alcoholic liver disease, reduces mtROS production, thereby inhibiting necroptosis [102]. In contrast, upon inhibition of mitophagy in renal tubular cells, necroptosis is induced via the ROS/RIPK3/MLKL signaling pathway [103]. Consequently, uncontrolled ROS can activate both mitophagy and necroptosis; however, mitophagy may mitigate necroptosis by eliminating excessive ROS.

On the other hand, RIPK3 also plays a significant role in the ability of necroptosis to regulate mitophagy. When RIPK3 undergoes phosphorylation due to damage, such as in ischemic/reperfusion (I/R) injury, it initiates the activation of Ca2+/Calmoduline-dependent protein kinase II (CaMKII), which, in turn, opens the MPTP [104]. At this stage, the preferred pathway for cell preservation and survival involves the activation of the RIP3-PGAM5-Drp1 pathway, which induces mitochondrial fission and mitophagy [105]. Studies confirmed that PGAM5 is an important regulator in PINK1-dependent mitophagy to protect cells from necroptosis [105]. However, under severe damage or certain stimuli, the pathway can shift towards necroptosis, where RIPK3 may reduce parkin phosphorylation, diminishing the interaction between parkin and LC3, thus halting mitophagy. Subsequently, phosphorylation of RIPK3 activates the RIPK3/RIPK1/MLKL necroptosis pathway. In this scenario, conducting RIPK3 knockdown will result in adenosine monophosphate-activated protein kinase (AMPK)/parkin-mediated mitophagy [106].

### Autophagy and mitophagy

Autophagy and mitophagy represent two facets of the same cellular process, occurring within cells and mitochondria, respectively. Both processes are orchestrated by the same machinery, albeit with slight differences regarding the preparation of mitochondria for autophagy. mTOR, one of the key regulators of autophagy, inhibits autophagy by halting ULK1 complex formation [21]. Studies have elucidated that mTOR activation also negatively affects mitophagy by halting the PINK1/ Parkin pathway [107]. As mentioned above, PINK1, has the ability to recruit autophagic receptors in parkin-independent way, including NDP52, and OPTN, activating both mitophagy and autophagy [34, 35]. So that, mitophagy and autophagy interconnection is mediated by PINK1 and ULK1 activity. Moreover, BNIP3, involved in mitophagy inhibits Ras homolog enriched in brain (Rheb), an upstream activator of mTOR, thus inhibiting mTOR, and activates autophagy [108]. Also, NIX or BNIP3 disrupt the attachment between beclin-1 and the anti-apoptotic BCL-2 family proteins, setting beclin-1 free and activate autophagy [109].

### Mitophagy and apoptosis

Regarding mitophagy and apoptosis crosstalk is intricately regulated by PINK1-Parkin-mediated mechanisms [110]. PINK1 contributes to apoptosis inhibition by phosphorylating key proteins, such as BCL-XL and BAD. While Parkin inhibits apoptosis by ubiquitinating pro-apoptotic proteins BAK and BAX. PINK1-Parkin-mediated mitophagy further contributes to apoptosis inhibition by other mechanisms, including upregulating BCL-2 and downregulating BAX. Additionally, selective removal of pro-apoptotic proteins, like phosphorylated p53, from mitochondria during mitophagy [110].

Conversely, the activation of Parkin has been shown to facilitate apoptosis, underscoring its dual role. This process involves the proteasomal degradation of myeloid cell leukemia-1 (MCL-1), a substrate of Parkin, which triggers apoptosis in response to mitochondrial damage [111]. Recently, Parkin has been shown to induce noncanonical apoptosis, independent of BAX/BAK by mediating the proteasomal degradation of the OMM. This process facilitates the release of cytochrome c into the cytosol, subsequently activating caspase-9 and triggering the apoptotic cascade [112]. However, later, upon the recruitment of the autophagic machinery to the OMM, the cell is protected from this form of cell death by eliminating the damaged mitochondria.

Notably, parkin is considered the point of intersection between mitophagy and apoptosis, as it participates in the ubiquitination of VDAC, where monoubiquitination halts apoptosis, while polyubiquitination activates mitophagy [113]. The way by which Parkin mediates these different ubiquitination processes is still under investigation. Moreover, the knockdown of peroxiredoxin 6 (PRDX6), an antioxidant enzyme, increases ROS, activates both mitophagy and apoptosis through the activation of PINK1/Parkin pathway [114]. Additionally, SIRT3 activates the AMPK/parkin-mediated mitophagy pathway and halts apoptosis [115]. Deleting uncoupling protein 2 (UCP2), an inner mitochondrial membrane protein, increases the expression of mitophagy-related proteins such as PINK1, beclin-1, and LC3, while reducing P62, thereby promoting mitophagy and apoptosis [116]. Finally, the impact of BNIP3 in interacting with LC3 stimulates mitophagy and apoptosis. However, when BNIP3 is silenced, it increases the expression of autophagy markers like lysosome-associated membrane glycoprotein 2 (LAMP2), the ratio of LC3-II/I and, beclin-1, thereby inhibiting apoptosis by enhancing general autophagy [117]. In summary, the link between mitophagy and apoptosis mainly depends on PINK1, Parkin, and its role in VDAC ubiquitination.

### Autophagy and apoptosis

Regarding the relationship between autophagy and apoptosis, they sometimes collaborate and sometimes oppose each other depending on the influencing factors and surrounding conditions [19]. At times, autophagy combines with apoptosis to induce cell death, such as in chemotherapy-treated cells. Conversely, autophagy plays a role in preserving cell viability and aiding in evasion from apoptosis, as observed in cancer treatment-resistant cells [118].

On the molecular basis, The ATG genes, which play a role in autophagy and autophagosome formation, have been found to be involved in apoptosis. For instance, Atg5 (molecular switch), when broken down by calpain, a calcium-activated protease, the resulting product, Atg5tN, enters the mitochondria and interacts with Bcl-2 proteins, releasing cytochrome c and inducing apoptosis. Similarly, ATG12 exhibits a critical function under conditions involving proteasome inhibition. The stabilization of non-degraded ATG12 enables its interaction with Bcl-2, which subsequently promotes the activation of BAX, ultimately initiating apoptosis. Additionally, Atg4D, normally inactive, when undergoes overactivation, is cleaved by caspase-3, resulting in cell death through both autophagy and apoptosis simultaneously. Atg3, an autophagic-protein-producing gene, contains a caspase-8 cleavage site and drives apoptosis [119]. Inhibition or deletion of Atg3 or Atg5 disrupts autophagosome formation, which, in turn, significantly reduces caspase-8 activation and the induction of apoptosis [120]. p62 binds and degrades Fas-associated phosphatase-1 (Fap-1), a negative regulator of Fas, inducing apoptosis [121]. Phosphatidylinositol-3-kinase (PI3K)/AKT/mTOR pathway is a shared inhibitory pathway between autophagy and apoptosis; where AKT is a key regulatory molecule that cleaves BAD, pro-apoptotic protein, inhibiting apoptosis. On the other hand, autophagy inhibition was attributed to mTOR inhibitory effect on ULK1 formation [122].

Regarding beclin-1, a key regulator of the autophagy process, it is considered a substrate for apoptotic caspases such as caspase-3, caspase-6, caspase-7, caspase- 8, and caspase-9. As a result, c-terminal cleaved beclin-1 is generated, which enters the mitochondria and releases cytochrome c, thereby inducing apoptosis and halting autophagy [123]. The expression level of beclin-1 regulates this dual action. Additionally, beclin-1 contains BH3 only domain, that can be cleaved by anti-apoptotic Bcl-2 or BCL-XL, inhibiting action of beclin-1 mediated autophagy, in parallel with stopping action of anti-apoptotic proteins, promoting apoptosis. This action can be resolved by phosphorylation of Bcl-2 or BCL-XL or ubiquitnation of beclin-1 [124, 125]. Notably, BAD or BH3 mimetic compounds, can also upregulate autophagy by competitively disrupting the interaction between Bcl-2 or BCL-XL and beclin-1 [126].

Moreover, AMPK activates apoptosis by suppressing the expression of anti-apoptotic Bcl-2 proteins [127]. In human colon cancer cell lines, AMPK early activation promoted autophagy by phosphorylating beclin-1, while late activation led to pronounced apoptosis by caspase-8-dependent cleavage of beclin-1. Interestingly, activation of apoptosis was significantly enhanced in ATG7-deficient cells, indicating that inhibition of autophagy sensitized cells to apoptosis [127]. P53 is known to activate apoptosis by stimulating pro-apoptotic proteins and inhibiting anti-apoptotic proteins. It has been found to play a role in autophagy, depending on its localization within the nucleus or cytoplasm, which varies based on the cellular context. Typically, p53 resides in the cytoplasm, but upon DNA damage, it translocates to the nucleus. Nuclear p53 activates autophagy via transcription modulation in the mTOR pathway [128]. Conversely, cytoplasmic p53 inhibits autophagy by interacting with FIP200, preventing ULK1 complex activation and thereby halting autophagy [119].

### Autophagy and ferroptosis

The relationship between autophagy and ferroptosis is somewhat puzzling because, in certain conditions, autophagy mediates the breaking down of iron storage proteins, ferritin, as a form of resource recycling and redistribution, by a process called ferritinophagy, thereby increasing iron overload and promoting ferroptosis. This was evident in a study that demonstrated an increase in cancer cells sensitivity to ferroptosis-inducing agents as erastin and RSL3, upon autophagic induction [129]. Similarly, erastin-induced ferroptosis was markedly attenuated in cells with knockouts or knockdowns of autophagy-related genes such as ATG5 and ATG7, due to a reduction in intracellular ferrous iron levels and lipid peroxidation [130]. Moreover, beclin-1 undergoes phosphorylation by AMPK and forms a complex with SLC7A11, thereby impeding its role in cystine transport, consequently leading to lipid peroxidation and ferroptosis [131]. Furthermore, cells exposed to the ferroptosis inducer compound Fin56 or to a high dose of copper, undergo autophagic degradation of GPX4, consequently activating ferroptosis [132, 133].

Additionally, p62 expression, crucial for autophagy completion [22, 134], has been found to play a role in activating ferroptosis. P62 prevents Nrf2 degradation and inhibit Keap1, thus leading to ferroptosis induction through p62/Keap1/Nrf2 pathway [135]. Autophagy modulates ACSL4 and consequently impacts sensitivity to ferroptosis. For instance, the autophagy receptor protein sequestosome 1 (SQSTM1) enhances advanced glycosylation end-product-specific receptor (AGER)-dependent ACSL4 expression, inducing ferroptosis [136]. Signal transducer and activator of transcription 3 (STAT3), a lysosomal modulator, triggers the expression and secretion of cathepsin B (CTSB) enhancing the permeability of lysosomal membranes [137]. However, over-expressed CTSB, leads to lysosomal dysfunction and promotes ferroptosis [138]. These findings point to a potential role of autophagy in ferroptosis, acting through lysosomal pathways. Lipophagy, an autophagic degradation of intracellular lipid droplets activates ferroptosis [139].

Conversely, other studies have shown that autophagy inhibits ferroptosis by eliminating oxidized lipids and damaged cells that stimulate ferroptosis. Paraoxonase 1 (PON1), a detoxifying enzyme, is upregulated during cell breakdown and recycling facilitated by autophagy, consequently increasing intracellular glutamate levels. This activates the antiporter to expel glutamate and, subsequently, cysteine influx, thus halting ferroptosis [140]. In autophagy-independent way, a study was conducted that upon subjecting cells to energy stress (low glucose), AMPK is activated, which partly inhibits ferroptosis [141].

On the other hand, ferroptosis interconnection with autophagy can be explained through action of upregulated ASCL4. Besides, ACSL4 involvement in inducing ferroptosis, it has the capacity to modulate intracellular autophagic signaling pathways, including AMPK and mTOR pathways. For instance, ACSL4, increases expression of angiotensin II, activating mitochondrial mTOR1/2 signaling proteins and fosters the proliferation of human H295R adrenocortical cells [142]. Acidic condition is required for the formation of autophagosomes and lysosomes, mediated by a V-ATPase, an ATP-dependent proton pump. ACSL4 has the capability to attach to V-ATPases, suppressing their activity and consequently impeding autophagosome formation [143].

### Necroptosis and autophagy

Regarding the relationship between necroptosis and autophagy, it can be summarized by the ability of RIPK3 to perform AMPK phosphorylation and activation. Consequently, the activated AMPK stimulates ULK1 and beclin-1, which are crucial autophagy regulators. Therefore, necroptosis activates early autophagy episodes [144]. This was confirmed, upon using necrostatin-1, a necroptosis inhibitor, the expression of LC3-II decreased, thus blocking autophagy [145]. Surprisingly, TNF-induced necroptosis, blocked autophagy late-stage, especially the autophagosome degradation with lysosomes [144]. This appears to be controversial, but scientists attributed this to the contribution of other dysregulated proteins in affecting the autophagosome- lysosome fusion. Also, MLKL was found to inhibit autophagy through its intracellular membrane association [146]. On the other side, autophagy can induce necroptosis through ROS, an autophagic byproduct [147]. Therefore, when using Baf-A1, an inhibitor of late-stage autophagy, there was a downregulation of RIP1, RIP3 necroptotic proteins, and a decrease in glutamate dehydrogenase expression level [148]. Similar outcomes were observed when blocking the early stage of autophagy with 3-MA suppressing RIP1/RIP3 interaction [149].

### Ferroptosis and apoptosis

A reinforcing relationship between ferroptosis and apoptosis was observed, where ferroptosis-inducing agents not only involved the generation of ROS and lipid peroxidation but also induced ER stress and unfolded protein response. ER stress activates the nuclear translocation and activation of C/EBP homologous protein (CHOP) pathway, which in turn activates PUMA and subsequently triggers apoptosis. This enhances cell death through two pathways, ferroptosis and apoptosis providing a potent approach for treating many diseases [150]. While p53, a known mediator in apoptosis, has been found to play a crucial role in activating ferroptosis by inhibiting SLC7A11 antiporter, and preventing GPX4 formation, consequently reducing glutathione levels and activating ferroptosis [151]. This theory is reinforced by a study demonstrating that increased expression of the inhibitor of apoptosis-stimulating protein of p53 (iASPP), which inhibits P53, halts ferroptosis as it activates the SLC7A11 antiporter [152]. Notably, the nuclear translocation of apoptosis inducing factor (AIF), induces both apoptosis and ferroptosis [153].

In a transcription-independent manner, p53 suppresses ferroptosis by interacting with dipeptidyl-peptidase-4 (DPP-4), a regulator of ferroptosis and lipid metabolism, supposing P53 dual action on ferroptosis regulation [154]. ACSL4, activates apoptosis by two mechanisms. ACSL4, stimulates the production of fatty acyl-CoA, thereby increasing oxidative stress. Consequently, intrinsic apoptosis is activated, due to the internal damage signals that accumulated in DNA and cell membrane [155]. Moreover, ACSL4 engages directly with and governs proteins implicated in apoptotic signaling pathways [156]. Conversely, ferroptosis-induced fatty acid oxidation, results in STAT3 activation, which in turn increases ACSL4 expression. This elevation enhances the integrity of the mitochondrial membrane and prevents apoptosis [157].

Although research demonstrated that the two pathways are interrelated, blocking one does not impede cell death but rather occurs through the other pathway. Treatment of germ cells with dihomogamma-linolenic acid, source of PUFA, resulted in cell death and sterility in normal cells as well as in apoptosis-deficient cells, confirming activation of ferroptosis cell death [158]. Furthermore, blocking ferroptosis with ferrostatin-1 (Fer-1) also led to germ cell death via apoptosis [159]. Erastin, known to stimulate ferroptosis, was also found to activate apoptosis, indicating that the pathways are interconnected [160]. GSH depletion, an important driver for ferroptosis, was found to suppress apoptosis, due to the proposed role of GSH in activating caspase-3 and caspase -8 [161].

### Apoptosis and necroptosis

Caspase-8 is a maestro orchestrating cell death, whether in its active or inactive form, through two different ways including, apoptosis and necroptosis. Under normal conditions, caspase-8 is activated in response to any harmful stimuli due to stimulation of TNFR/Fas. Caspase-8 then activates caspase-3, inducing apoptosis [162]. In this scenario, necroptosis inhibition is attributed to the ability of active caspase-8 to cleave the RIPK1, RIPK3, and CYLD, key substrates of necroptosis, thereby preventing necroptosis [163]. Notably, RIPK1 kinase activation is mediated by CYLD-dependent deubiquitination, leading to necroptosis initiation [164].

Caspase-8 interacts with FLICE-like inhibitory protein (c-FLIP), an inactive caspase-8 homolog, to regulate apoptosis and necroptosis. While caspase-8 homodimers primarily drive apoptosis, caspase-8/ c-FLIP_L_ heterodimers suppress both apoptosis and necroptosis, promoting cell survival by reducing caspase-8 enzymatic activity. This partial activity is sufficient to cleave and inactivate RIPK1 and RIPK3, thereby inhibiting necroptosis. However, in cells with high RIPK3 expression, necroptosis can be induced if basal caspase-8 activity is inhibited, as seen in TNF signaling [163]. Also, the inhibition of caspase-8 by z-VAD-fmk or viral c-FLIP mimetics causes a switch to the necroptosis pathway [165]. The embryonic lethality in caspase-8 or FADD-deficient mice is fully rescued by the knockout of RIPK3, MLKL, or RIPK1 [166–170]. Interestingly, the formation of heterodimers between caspase-8 and the shorter c-FLIP isoform, c-FLIP_S_, inhibits caspase-8 activity but enhances complex II assembly, inhibiting apoptosis and promoting necroptosis [171, 172].

RIPK1 governs cell fate through its dual roles as an adaptor and kinase, interacting with RIPK3 and caspase-8 to orchestrate a finely tuned balance between cell survival, apoptosis, and necroptosis. RIPK1 adaptor function promotes cell survival by activating NF-κB and MAPK pathways, and inhibiting cell death by preventing TNF-induced apoptosis and links RIPK3 to caspase-8, suppressing necroptosis [163, 173, 174]. Perinatal lethality was observed in RIPK1-deficient mice which results from simultaneous, uncontrolled activation of caspase-8 and RIPK3. While mice lacking RIPK1, caspase-8, and RIPK3 were able to survive to adulthood [175–177]. Moreover, RIPK1-deficient mice develop severe inflammatory bowel disease due to extensive caspase-8-driven apoptosis within the intestinal epithelium, resulting in early death [178].

Conversely, RIPK1 possesses an enzymatic activity that has two different modulating roles on RIPK3, promoting cell death, particularly when cIAP1/2 or TAK1 is degraded [179]. RIPK1’s kinase activity drives TNF-induced necroptosis by activating RIPK3 but also limits excessive RIPK3 activity in interferon (IFN)- and TRIF-driven signaling by facilitating caspase-8-mediated necroptosis inhibition. Notably, the order of RIPK1 and RIPK3 kinases plays an important role in the type of cell death mechanism [163]. Unlike RIPK1-null mice, which die shortly after birth, mice with a kinase-inactive RIPK1 mutation are viable, underscoring that RIPK1’s adaptor function, rather than its kinase activity, is crucial for survival postnatally. Further, these kinase-dead mice are fully resistant to TNF-induced shock and TNF-mediated cell death, indicating that RIPK1 kinase activity is not essential for the activation of MAPK and NF-κB pathways but is a primary driver of apoptosis and necroptosis in the TNF-shock response [163]. Moreover, recent research shows that the death domain of RIPK1 restrains ZBP1- and TRIF-mediated cell death [180].

RIPK3 is a key regulator that can influence both necroptosis and apoptosis. Similar to RIPK1, RIPK3 possesses an adaptor activity and a kinase activity. Typically, RIPK1 activates RIPK3 to drive necroptosis in response to TNF receptor signaling, utilizing RIPK3 kinase activity. However, if RIPK3’s kinase activity is disrupted as in the D161N kinase-dead RIPK3 mutant, it switches to promote apoptosis through its adaptor function. Although the exact mechanism is still unclear, it has been proposed that the D161N mutation changes the conformation of RIPK3, potentially exposing its RHIM domain, a region crucial for protein-protein interactions, triggering apoptosis instead. Thus, RIPK3’s kinase domain seems to act as a protective “mask” over the RHIM to prevent unintended apoptosis under normal conditions [181].

Moreover, it was observed under conditions where apoptosis is mediated by RIPK1, RIPK3 has been shown to enhance the complete activation of caspase-8 downstream of TNFR1 signaling, independently of both its kinase activity and an intact RHIM domain [182]. However, in the case of RIPK1’s absence, TNF can activate RIPK3, resulting in high cellular levels and promoting necroptotic cell death [183]. The balance of RIPK3’s influence on apoptosis versus necroptosis is finely regulated to prevent excessive cell death. If the necroptosis pathway is blocked, for instance, by inhibiting RIPK3 or MLKL, cells may switch to apoptosis as a secondary pathway, but the timing and dynamics of cell death are altered [184]. This interdependency means RIPK3 functions as a versatile control point, ensuring that cells can switch between apoptosis and necroptosis based on specific molecular signals and conditions.

The interconnection between apoptosis and necroptosis includes the action of Drp1, which have got a role in maintaining apoptosis through Bax/Bak apoptotic pathway or BNIP3- mediating apoptosis [43]. Similarly, PGAM5 mediates Drp1 activation, mediating necroptotic cell death [43]. Some studies exclude the importance of PGAM5 in activating Drp1, so more research is required to understand the exact mechanism.

### Pyroptosis and apoptosis

Regarding the interconnection between pyroptosis and apoptosis, studies have demonstrated that pyroptosis has the capability to activate the apoptosis process through caspase-1 mediated cleavage of BID, a pro-apoptotic protein in I/R injury model [185]. In gasdermin-D deficient cells, caspase-1 drives the apoptotic pathway through the BID/caspase-9/caspase-3 pathway, which can be followed by GSDME-dependent pyroptosis to eliminate uncleared apoptotic bodies [186]. This was supported by many studies, including a recent finding in the context of hepatitis C virus infection, showing that GSDMD knockout resulted in enhanced activation of caspase-3 [187]. In the absence of GSDMD, apoptosis is induced in LPS plus nigericin-treated or *S. typhimurium*-treated mouse macrophages depending on NLRP3 activation and not caspase-1 [188]. Notably, inflammasome-induced apoptosis may represent a physiologically relevant cell death mechanism in cell types with minimal or absent GSDMD expression, such as neurons and mast cells.

Additionally, in human neuronal cells subjected to serum deprivation in vitro, caspase-1 activation was induced, subsequently cleaving and activating caspase-6, an apoptotic caspase. This led to the activation of downstream apoptotic pathways and, ultimately, cell death [189]. Furthermore, caspase-1 can activate caspase-7, upon inflammasome activation by Salmonella infection, as well as during activation by LPS + ATP stimulation, promoting pyroptosis. While this activation is absent in caspase-1 deficient macrophages, it switches to the apoptosis pathway instead [190]. Notably, PARP1, a hallmark of apoptosis, is a downstream target of activated caspase-1 and caspase-7. Therefore, caspase-1 activity is essential for shifting the cellular death pathway from apoptosis to pro-inflammatory pyroptosis [191]. Remarkably, although canonical inflammasomes and caspase-1 primarily drive pyroptosis by activating GSDMD, they also trigger the activation of caspase-3 and caspase-7, which inactivate GSDMD and suppress pyroptosis. The slower activation of these caspases suggests a regulatory feedback mechanism, where they dampen pyroptosis after its initiation. This dual function ensures the effective elimination of infected cells while preventing excessive inflammation [192]. Moreover, caspase-11, a non-canonical pyroptotic molecule has a dual role in activating apoptosis. Specifically, it activates pro-caspase-3 and caspase-1 in pathological conditions, inducing apoptosis both directly and indirectly, respectively [193].

Caspase-8 and FADD serve as key regulators of cell fate, determining whether cells undergo non-inflammatory apoptosis or inflammatory pyroptosis. Caspase-8 is a key initiator of apoptosis. When activated, it directly triggers the apoptotic pathway by cleaving and activating downstream executioner caspases, such as caspase-3 and caspase-7. These caspases degrade cellular components, leading to controlled cell death without inflammation. Caspase-1-deficient cells compensate for the absence of pyroptosis by initiating apoptosis through caspase-8-dependent pathways, such as those triggered by NLRC4 and AIM2 inflammasome activation. This process involves the recruitment of FADD, ASC, and the activation of caspase-8, leading to caspase-3 activation and apoptotic cell death. Interestingly, caspase-8 also indirectly participates in pyroptosis by promoting NLRP3 inflammasome activation through NF-κB priming and post-translational modifications [194]. Moreover, caspase-8/c-FLIP complex activates NLRP3 inflammasome [195]. In macrophages exposed to salmonella infection, caspase-8 contributes to the maturation of pro-IL-1β, influencing inflammation rather than pyroptotic death [196]. Caspase-8 also cleaves GSDMD, promoting pyroptosis under certain conditions, including TAK1 inhibition [197]. Additionally, caspase-8’s inactivation can drive pyroptosis by forming ASC specks, further implicating it as a critical switch between apoptosis and pyroptosis [198].

FADD functions as an adaptor protein that facilitates the activation of caspase-8 by forming a DISC with caspase-8, which leads to caspase-8-mediated activation of executioner caspases, promoting apoptosis. FADD also contributes to pyroptosis by regulating inflammasome activation. It promotes the activation of the NLRP3 inflammasome and caspase-1, leading to pyroptotic cell death [199]. Additionally, FADD interacts with RIPK1 and caspase-8 in the ripoptosome to enhance caspase-8-mediated GSDMD cleavage, further facilitating pyroptosis [200]. Hence, FADD acts as a coordinator of cell death pathways by modulating caspase-8 activity.

Intrinsic apoptosis, involving BAX/BAK-mediated mitochondrial destabilization, activates caspase-8 in a feedforward loop that links to IL-1β release and inflammasome activation [201]. The precise interplay between intrinsic apoptosis and NLRP3 activation, especially in terms of GSDMD cleavage, remains an area of ongoing investigation. As NLRP3 activation mediated IL-1β maturation appears to occur through K+ efflux, independent of GSDMD. Instead, apoptotic caspases, including caspase-3 and caspase-7 inactivate GSDMD by cleaving it at aspartate 88 [192, 202]. However, it was observed that other pore-forming molecules might participate in this interplay, as the activation of pannexin-1 following caspase-8 or caspase-9 activation, triggering NLRP3 inflammasome assembly via a process independent of GSDMD and GSDME [203]. There is evidence indicating that the APAF1-apoptosome engages with caspase-11 under conditions of bile acid challenge within cells. This interaction results in the induction of pyroptotic death through a GSDME-mediated mechanism [204]. Nevertheless, the regulatory mechanisms for switching between the APAF1/caspase-11 pyroptosome and APAF1/caspase-9 apoptosome remain elusive.

Interestingly, caspase-3/GSDME, act as a switch mechanism between apoptosis and pyroptosis during chemotherapy, depending on the level of GSDME expression. Caspase-3 cleaves highly expressed GSDME in cancer cells, releasing the N-terminal domain which forms pores, mediating pyroptosis. Conversely, GSDME acts as an upstream activator for caspase-3 in cases of low expression status, activating apoptosis [205]. Nonetheless, the interaction between caspase-3 and GSDMD differs, as caspase-3 cleaves the active GSDMD-N, inhibiting pyroptosis [192, 203].

Overall, these studies indicate that apoptotic caspases, including caspase-8 and caspase-3, can modulate pyroptosis in cells expressing GSDME and GSDMD. This suggests that the nature of cell death is determined not solely by the activated caspases but by the presence of specific caspase substrates that facilitate either apoptotic or pyroptotic cell death pathways.

### Ferroptosis and necroptosis

Cysteine is an amino acid that serves as an intersecting point between ferroptosis and necroptosis. Cysteine acts as a ROS sensor, found in RIP1. Upon increasing mtROS, cysteine drives RIP1 autophosphorylation. Phosphorylated RIP1 can then recruit RIP3, forming the necrosome complex [206]. On the other hand, cysteine which is converted to cystine in most tissues, enters cells with the assistance of the SLC7A11 antiporter in exchange for the efflux of glutamate. This process results in glutathione biosynthesis and inhibition of ferroptosis [207]. Interestingly, when the antiporter is blocked, ROS increases, triggering a double death mechanism involving both ferroptosis and necroptosis in hepatocellular carcinoma cells [208]. Moreover, knocking down of progranulin, a cysteine-rich growth factor, increases ROS and promotes necroptosis in I/R injury mice model [209]. Similarly, upon GPX4 overexpression, both ferroptosis and necroptosis were inhibited due to decreasing ROS [85].

Additionally, the heat shock protein 90 (HSP90) plays a role in regulating both necroptosis and ferroptosis. HSP90 assists in the formation of the RIP1/RIP3 complex, thereby facilitating necroptosis [210]. This mirrors the effect of HSP90 on ferroptosis, where HSP90 degrades GPX4, consequently activating ferroptosis [211]. This relationship has been demonstrated through the use of tanespimycin (17-allylamino-17-demethoxygeldanamycin), an HSP90 inhibitor, which, according to the molecular mechanism described, blocks both types of cell death [212]. Similarly, in a recent study, overexpression of HSP90 due to cerebral I/R injury, activates both necroptosis and ferroptosis pathways via the HSP90-GCN2-ATF4 pathway [213]. Where GCN2 (general control nonderepressible 2) is a protein kinase that senses nutrient deficiency, while ATF4 stands for activating transcription factor 4, which has a role in the transcription of many genes as well as regulates metabolic and oxidative homeostasis in tissues.

It is known that iron overload activates the ferroptosis pathway. However, what is new is that it also activates necroptosis. Iron overload increases ROS, which stimulates the opening of mitochondrial pores, releasing mtROS, and consequently activating necroptosis [214]. Moreover, a recent study reported that necrostatin-1, a potent inhibitor of RIPK1-dependent necroptosis, attenuates ferroptosis triggered by erastin or sulfasalazine in Huh7 and SK-HEP-1 cells. Its ferroptosis-inhibitory effect is hypothesized to involve the upregulation of xCT expression, counteracting system xc− inhibition in these cell lines. More research is needed to understand the precise mechanism [215].

It was found that the relationship between necroptosis and ferroptosis is not only interconnected but also alternative. Scientists have proposed an explanation that this could be attributed to membrane lipid composition. Both necroptosis and ferroptosis lead to alterations in cell membrane, and ferroptosis is primarily dependent on membrane lipid metabolism. It was assumed that MLKL may cause PUFA depletion, thus halting ferroptosis. On the other hand, ACSL4 is a crucial enzyme involved in arachidonic acid insertion into membrane phospholipids, thereby influencing lipid metabolism and ferroptosis. ACSL4 overexpression, not only downregulates GPX4, but also, makes the cell membrane less responsive to the pore-forming MLKL and consequently inhibits necroptosis [216]. Notably, ACSL4 knock down, inhibits ferroptosis, and activates necroptosis [217].

### Necroptosis and pyroptosis

MLKL, is a necroptotic molecule that induces pyroptosis, that was discovered upon observing ASC oligomerization inhibition in cells lacking MLKL [218]. The effect of MLKL on pyroptosis can be attributed to the potassium efflux that happened as a result of MLKL pore formation, which was found to trigger inflammasome activation, driving RIPK3/MLKL/NLRP3 pathway [219, 220]. An intriguing finding from a recent report highlights that necrosulfonamide, a well-known MLKL inhibitor used to prevent necroptosis in human cells, also functions as an inhibitor of GSDMD, inhibiting pyroptosis [221]. Interestingly, RIPK3 has the ability to activate NLRP3 in the absence of MLKL [222]. Upon activation of ZBP1 protein by endogenous or viral nucleic acid ligands, RIPK3 and caspase-8 are recruited. This recruitment results in the activation of NLRP3 inflammasomes, leading to pyroptosis [223, 224]. Similarly, as we mentioned above, the effect of HSP90 inhibition on abolishing necroptosis. A neuroprotective effect was obtained after subarachnoid hemorrhage in rats using HSP90 inhibitor, as pyroptosis is inhibited through HSP90/RIP3/NLRP3 pathway [225].

### Ferroptosis and pyroptosis

Ferroptosis plays a role in activating pyroptosis, where lipid peroxidation, a key factor in ferroptosis, activates the cleavage and activation of GSDMD. Consequently, upon inhibition of GPX4, an increase in lipid peroxidation occurs, which activates GSDMD cleavage and subsequently triggers pyroptosis [226]. The Nuclear factor erythroid 2-related factor 2 (Nrf2) pathway is primarily a regulatory pathway controlling oxidative stress within the cell, this pathway normally regulated by Kelch-like ECH-associated protein 1 (KEAP1) [227]. NrF2 oversees the transcriptional control of three principal categories of ferroptosis-associated genes: iron metabolism genes, including FTH1, HO-1, and FTL; ROS metabolism genes such as PPARG; and genes involved in regulating GSH synthesis like GPX4 and SLC7A11 [228]. Studies have shown the overexpression of Nrf2 or KEAP1 knockdown leads to increased expression of HO-1 and GPX4, consequently enhancing GSH synthesis. This leads to the inhibition of the ferroptosis pathway [229]. Conversely, Nrf2 stimulates NLRP3 and AIM, thereby promoting cell death via pyroptosis. Notably, if Nrf2 succeeds in reducing ROS levels, it can inhibit the pyroptosis pathway as well [230].

### Pyroptosis and autophagy

Regarding the relationship between pyroptosis and autophagy, it is inverse, where the activation of the pyroptosis pathway leads to the suppression of autophagy. Pyroptosis–mediating autophagy impairment in high glucose-primed macrophages was attributed to increasing ROS. Where the ROS, activates P62 overexpression and mTOR phosphorylation, in addition to reducing beclin-1, LC3-II level, and impedes autophagosome maturation [23]. Moreover, the activation of p62 stimulates the Nrf2/ antioxidant response element (ARE) axis, which activates pyroptosis in ox-LDL-treated macrophages [231]. Conversely, activation of the autophagy pathway reduces ROS levels, thereby suppressing the pyroptotic pathway [232]. Moreover, AMPK/SIRT1, an autophagic pathway has a profound impact on inhibiting pyroptosis. In a study, colchicine activates autophagy by stimulating AMPK, which in turn upregulates SIRT1, crucial for inflammation control through histone deacetylation. Consequently, it weakens the inflammation, alleviates oxidative stress mediating the pyroptosis pathway, and halts the entire pyroptotic process [19, 233]. Recently, Peroxiredoxin 1, an antioxidant enzyme, has been found to be upregulated in oral squamous cell, and has played a great role in the crosstalk between pyroptosis and autophagy [234]. Thus, pyroptosis is a negative regulator of autophagy and vice versa [234, 235].

However, a recent study revealed that autophagy has the capability to activate pyroptosis through CTSB found in lysosomes, which emerge during the autophagy process. In MCF-7 breast cancer cells, activation of autophagy by curcumin resulted in the upregulation of CTSB, consequently activating NLRP3/caspase-1/GSDMD pathway, thereby halting the proliferation and inducing the pyroptotic cell death of cancer cells [236]. Similarly, in an in vitro study using diabetic cardiomyopathy mice model [237]. Recently, a study confirmed that inhibiting autophagy, results in halting the progression of hyperuricemic nephropathy by inhibiting CTSB/NLRP3 pathway-mediating pyroptosis [238]. Due to the contradiction of these findings with most of the existing research, further experiments are warranted on various disease types and in vivo studies to elucidate the interplay between these pathways.

Since ferroptosis, necroptosis, and pyroptosis are all considered lytic cell death mechanisms that lead to the release of cellular contents and DAMPs [239]. It can be proposed that DAMPs released from ferroptosis may act as a destruction signal triggering necroptosis or pyroptosis, further research need to be done to confirm this point of view.

## Conclusion and future perspectives

Death is a term with negative connotations, as it signifies the loss of a person or valuable entity. However, at the cellular level, the situation differs, as death can be beneficial, during development or to achieve homeostasis. However, exceeding or falling below normal levels, may lead to diseases. Various types of cell death have been extensively researched, including PCD, such as apoptosis, autophagy, mitophagy, and others classified as inflammatory cell death like pyroptosis, necroptosis, as well as unprogrammed cell death like necrosis. Other types have also been elucidated as ferroptosis, enriching our understanding of the subject and potentially impacting the treatment of many challenging diseases.

Based on our thorough understanding of the molecular mechanisms of autophagy, mitophagy, apoptosis, ferroptosis, necroptosis, and pyroptosis, it is critical to put these into the context of tissue homeostasis and pathology. At first glance, apoptosis appears to be the preferred mechanism for cell death over inflammatory PCD to avoid inflammation or the death of many cells. However, the truth is that each type of cell death has its role in the way it was created. Apoptosis results in the death of a few cells locally, which is beneficial in cases of minor damage. However, if many cells are affected, alternative types of cell death are required.

While those mechanisms are significantly different, there is coexistence and mutual crosstalk among them. Indeed, those six pathways, constitute a cohesive and coordinated cell death unit, in which one pathway can activate, inhibit or flexibly compensate for the other. Although this crosstalk offers opportunities for developing novel therapeutic approaches targeting cell death pathways to treat diverse diseases. But, these interactions, render the specificity of molecular markers used to differentiate cell death types is becoming less clear.

Cell death mechanisms can be expressed as slicing meat with a knife, where the instrument used plays a pivotal role in determining the final outcome of the meat and its texture. Using more than one tool can be used to slice more effectively and efficiently in case of cancerous, diseased, or non-functional cells, inducing one or multiple types of cell death mechanisms. Reciprocally, we can employ a knife guard to prevent it from cutting the meat, inhibiting cell death mechanisms. Hence, our forthcoming objective may necessitate an integrated perspective to comprehend more types, addressing their specificity and their crosstalk, understanding their roles in disease development, and establishing novel targeted therapeutic interventions.

According to our knowledge, this is the first review addressing the crosstalk among the six-cell death pathways, in addition to focusing on the impact of the important regulators within each pathway on one another. This opens our minds to more and more relationships, and each new relationship discovered is considered a new weapon for treating either a new disease or an old resistant one.
